# Metabolic light absorption, scattering, and emission (MetaLASE) microscopy

**DOI:** 10.1126/sciadv.adl5729

**Published:** 2024-10-18

**Authors:** Brendon S. Restall, Nathaniel J. M. Haven, Matthew T. Martell, Brendyn D. Cikaluk, Joy Wang, Pradyumna Kedarisetti, Saymon Tejay, Benjamin A. Adam, Gopinath Sutendra, Xingyu Li, Roger J. Zemp

**Affiliations:** ^1^ Department of Electrical and Computer Engineering, University of Alberta, 116 Street & 85 Avenue, Edmonton, Alberta T6G 2R3, Canada.; ^2^ Department of Medicine, Faculty of Medicine & Dentistry, University of Alberta, Edmonton, Alberta, Canada.; ^3^ Department of Laboratory Medicine and Pathology, University of Alberta, 8440-112 Street, Edmonton, Alberta T6G 2B7, Canada.

## Abstract

Optical imaging of metabolism can provide key information about health and disease progression in cells and tissues; however, current methods have lacked gold-standard information about histological structure. Conversely, histology and virtual histology methods have lacked metabolic contrast. Here, we present metabolic light absorption, scattering, and emission (MetaLASE) microscopy, which rapidly provides a virtual histology and optical metabolic readout simultaneously. Hematoxylin-like nucleic contrast and eosin-like cytoplasmic contrast are obtained using photoacoustic remote sensing and ultraviolet reflectance microscopy, respectively. The same ultraviolet source excites endogenous Nicotinamide adenine dinucleotide (phosphate), flavin adenine dinucleotide, and collagen autofluorescence, providing a map of optical redox ratios to visualize metabolic variations including in areas of invasive carcinoma. Benign chronic inflammation and glands also are seen to exhibit hypermetabolism. MetaLASE microscopy offers promise for future applications in intraoperative margin analysis and in research applications where greater insights into metabolic activity could be correlated with cell and tissue types.

## Teaser

MetaLASE acquires simultaneous metabolic and virtual histology images in freshly resected tissues using ultraviolet light.

## INTRODUCTION

Assessment of cellular-level metabolism is critical for both diagnosing and better understanding certain diseases such as cancer, fatty liver disease, autoimmune disorders, pancreatitis, and more. Optical metabolic imaging using the optical redox ratio (ORR) has proven to be a powerful means of tracking metabolism. However, despite advances in optical metabolic imaging techniques, existing methods are unable to simultaneously acquire high-resolution, coregistered metabolic and hematoxylin and eosin (H&E)–like histological contrast. Simultaneous acquisition is important for faster imaging speeds, increasing the potential utility of such a multimodal system in time-constrained settings such as intraoperative margin assessment during tumor resection surgeries. A high-resolution metabolic and virtual histology output is critical to ensure that the imaging modality is capable of observing cellular-level metabolic changes, with corresponding morphological information at a resolution equivalent to or better than that provided by the current H&E-stained gold standard.

To address these unmet needs, we introduce metabolic light absorption, scattering, and emission (MetaLASE) microscopy, which incorporates multiple microscopy techniques simultaneously using a single ultraviolet (UV) excitation laser, with pulse energies as low as 350 pJ. These include (i) UV confocal reflectance microscopy to capture UV scattering contrast, (ii) autofluorescence microscopy to capture information about molecules relevant to tissue metabolism as well as structural molecules such as collagen, and (iii) UV photoacoustic remote sensing (UV-PARS) microscopy, which captures UV absorption contrast. All modalities are acquired in reflection mode. The plethora of data from MetaLASE microscopy enables acquisition of images with strong similarity to histology but without fixing or staining protocols and concurrently enables snapshots of metabolic activity in cells and tissues. Unlike many transmission-based microscopy methods, the reflection-mode capabilities of MetaLASE mean that thick tissues can be imaged, enabling applications including imaging of surgical resection specimens. Thick tissue imaging is accomplished by mounting tissues beneath a UV-transparent glass coverslip and rapidly scanning the tissues using a fast-scanning stage system. All the submodalities of MetaLASE may be captured simultaneously at a rate of 7 min/cm^2^ with spatial resolution as fine as 273 nm for UV-PARS. Micrometer-scale or submicrometer-scale resolution is obtained with all submodalities. The field of view in the fast-scan direction is currently limited to 1.2 cm by the stage travel range but can be extended in the slow-scanning direction to as large as needed. MetaLASE is currently limited to imaging only the superficial surfaces of tissues to depths limited to tens of micrometers but offers confocal sectioning ability with micrometer-scale depth of focus.

Nonoptical metabolic imaging methods typically rely on metabolite imaging and glucose analogs such as fluorodeoxyglucose (*1*). Most optical metabolic microscopy methods have relied on autofluorescence emission from two metabolic electron carrier molecules of interest: Nicotinamide adenine dinucleotide (phosphate) (NAD(P)H) and flavin adenine dinucleotide (FAD) (*2*, *3*). The ORR, defined as the ratio of FAD autofluorescence intensity to the sum of NAD(P)H and FAD autofluorescence intensities (*4*, *5*), has emerged as a robust means of imaging metabolic state. This is a valuable metric that can indicate metabolic changes in normal tissues and pathological processes including neurodegeneration, immune response, and cancer (*6*). In malignant samples, monitoring metabolic state in addition to tissue morphology can provide further insight into the tumor phenotype, which is useful for predicting aggression, treatment response, and prognosis (*2*, *7*, *8*).

A number of macroscopic optical metabolic imaging methods have been demonstrated based on NAD(P)H and FAD autofluorescence, including the Chance redox scanner, fast-clearing ultramicroscopy, and autofluorescence endoscopy (*5*, *9*–*13*). Microscopic metabolic imaging methods have included multiphoton excitation of endogenous chromophores (*14*), UV-excited autofluorescence microscopy (*15*, *16*), and fluorescence lifetime imaging microscopy (*14*). While macroscopic methods have afforded whole-tissue evaluation, resolution is insufficient to investigate microscale histological features or evaluate cellular energetics. In contrast, microscopic methods have often been limited in the field of view, precluding whole-tissue context, and have still lacked histological realism for pathologist interpretation.

Histological information has been achieved with optical virtual histology methods, but most current methods cannot yet achieve metabolic imaging simultaneously, despite some promising recent progress (*17*). Previous virtual histology methods are outlined in Table 1 and reviewed further in the Supplementary Materials. Reflection-mode operation and label-free contrast are desirable characteristics so that fresh, thick tissue imaging can be performed without contaminating or modifying the tissue, ensuring subsequent gold-standard histological analysis can be performed. However, most existing label-free microscopy methods have lacked positive nuclei contrast. Haven *et al.* first demonstrated UV-PARS microscopy as a means to provide positive nuclei contrast in reflection mode (*18*, *19*). PARS microscopy is used to obtain absorption contrast in a label-free, noncontact configuration, using a nanosecond-pulsed excitation laser to generate absorption-induced reflectivity modulations in a precisely cofocused interrogation beam (*20*–*22*). When a ~266-nm excitation source is used, PARS provides cell nuclei–specific contrast (*18*, *19*, *23*–*25*). In addition, complementary cytoplasmic contrast has been achieved simultaneously using various approaches including measurement of an optical back-scattering signal, PARS imaging with additional cytochrome-targeted excitation wavelengths, and frequency and time-domain decomposition of PARS signals (*26*–*29*). The all-optical implementation of PARS has further facilitated its combination with other optical imaging modalities such as optical coherence tomography and fluorescence microscopy for additional contrasts (*30*–*33*). Maximally realistic virtual H&E histology was achieved using both UV absorption and UV scattering contrast as described by Haven *et al.* (*34*), and cycle-consistent generative adversarial network (CycleGAN)–based stain style transfer as demonstrated by Martell *et al.* (*35*). In a recent work, we also validated these PARS virtual histology approaches in a pathologist reader study. For the task of identifying malignancy, pathologists viewing virtual histology images achieved sensitivity and specificity of 0.96 and 0.91 in breast tissues and 0.87 and 0.94 in prostate tissues, respectively (*35*). In addition, pathologists scored the quality of virtual histology images to be superior to frozen sections, known to be prone to staining artifacts (*35*). However, PARS virtual histology has not yet been combined with complementary metabolic contrast.

**Table 1.** Comparison of MetaLASE with existing metabolic and virtual histology imaging modalities. Unlike many approaches in the literature, our virtual histology approach is label-free and, unlike many virtual H&E microscopy implementations, our MetaLASE approach offers metabolic information. Note that specifications represent reported system implementations but not necessarily fundamental limitations of each technology. 2P, two-photon; 3P, three-photon; SHG, second harmonic generation; THG, third harmonic generation; NLM, nonlinear microscopy; CFM, confocal fluorescence microscopy; SIM, structured illumination microscopy; FLIM, fluorescence lifetime imaging microscopy; TA-PARS, total absorption PARS; MUSE, microscopy with UV surface excitation; LSM, light sheet microscopy; SRS, stimulated Raman scattering; PAM, photoacoustic microscopy; MS-DUV, multispectral deep UV; RCM, reflectance confocal microscopy.

Metabolic microscopy						
Modality	Lateral resolution (μm)	Scan rate (min/cm^2^)	Contrast mechanism	Target molecules	Simultaneous histological contrast	References
**MetaLASE**	0.27	7	Nonradiative absorption, scattering, autofluorescence	NAD(P)H FAD	✓	This work
NLM	<0.5	13.5	2PAF, 3PAF, SHG, THG	NAD(P)H	×	(*2*, *7*, *8*, *62*–*64*)
				FAD		
				FMN		
				Collagen		
CFM	0.7	4	Autofluorescence	NAD(P)H	×	(*65*, *66*)
				FAD		
SIM	1.3	0.23	Autofluorescence	NAD(P)H	×	(*67*, *68*)
				FAD		
FLIM	<0.5–500	0.75–33.7	Fluorescence lifetime	NAD(P)H	×	(*14*, *69*, *70*)
				FAD		
SRS	0.3	11.4	Inelastic scattering	Macromolecules	×	(*71*, *72*)
				+/− Deuterium		
				Labeling		
Virtual histology						
Modality	Lateral resolution (μm)	Scan rate (min/cm^2^)	Contrast mechanism	Label-free	Simultaneous metabolic contrast	References
**MetaLASE**	1.0	7	Nonradiative absorption, scattering, autofluorescence	✓	✓	This work
**UV-PARS**	0.39	7	Nonradiative absorption	✓	×	(*34*, *35*, *60*, *73*)
**TA-PARS**	0.35	667	Nonradiative absorption, scattering, autofluorescence	✓	×	(*53*, *74*–*76*)
**MUSE**	0.6	0.9–1.3	Fluorescence	✓/×	×	(*77*)
**LSM**	1.5–1.8	0.2	(Auto)fluorescence	✓/×	×	(*78*, *79*)
**NLM**	0.44	8.9	2P, 3P, SHG, THG	×	×	(*62*, *64*, *80*–*82*)
**SRS**	0.36	200	Inelastic scattering	✓	×	(*83*, *84*)
**UV-PAM**	0.33–1.6	14.2–1000	Nonradiative absorption	✓	×	(*85*–*88*)
**SIM**	1.25–2	0.25–1.7	(Auto)fluorescence	✓/×	×	(*67*, *89*)
**MS-DUV**	0.3	17	Absorption, scattering	✓	×	(*90*)
**CFM**	0.75	1.25	(Auto)fluorescence	✓/×	×	(*91*–*93*)
**RCM**	6.25	2	Scattering	✓	×	(*91*–*93*)

**Fig. 1.**
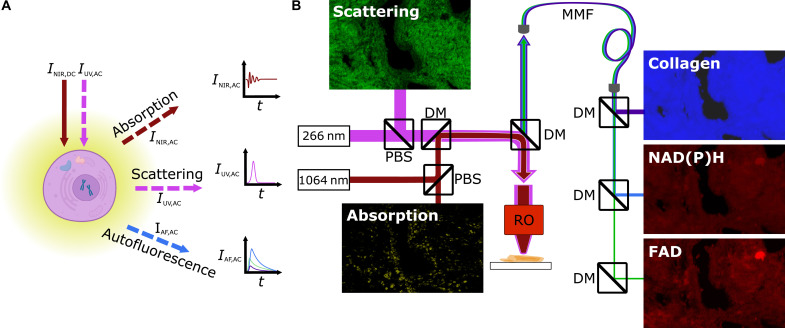
MetaLASE contrast mechanisms and simplified system diagram. (**A**) Absorption, scattering, and autofluorescence emission contrast mechanisms and (**B**) imaging system. *I*_NIR,DC_, incident continuous-wave NIR interrogation intensity; *I*_UV,AC_, incident pulsed UV excitation intensity; *I*_NIR,AC_, back-scattered absorption-induced modulated NIR interrogation intensity; *I*_UV,AC_, back-scattered pulsed UV excitation intensity; *I*_AF,AC_, autofluorescence emission; *t*, time; PBS, polarizing beam splitter; DM, dichroic mirror; RO, reflective objective; MMF, multimode fiber.

MetaLASE microscopy is a functional extension to UV-PARS that simultaneously achieves both virtual H&E histology and metabolic contrast from endogenous chromophores as shown in Fig. 1A. This offers comprehensive, coregistered structural and functional information in a single scan of the sample. A simplified system diagram is shown in Fig. 1B, with a more detailed system outline provided in fig. S2. We use two wavelengths: a nanosecond-pulsed 266-nm laser and a continuous-wave 1064-nm broadband superluminescent diode source, both far removed from the desired autofluorescence detection bandwidths. The two beams are combined and cofocused using a reflective objective. The back-scattered 266-nm light is used to approximate eosin contrast for virtual histology. The modulated component of the 1064-nm light is the PARS signal and represents optical absorption of the 266-nm excitation beam. It is used as a virtual hematoxylin channel. Autofluorescence emission simultaneously excited by the 266-nm pulsed laser is split into three channels, representing the signal predominantly from collagen, NAD(P)H, and FAD, as illustrated in fig. S1.

We demonstrate MetaLASE microscopy for imaging formalin-fixed paraffin embedded (FFPE)–sectioned tissues, live cell cultures, and freshly resected tissue specimens. Experiments are designed to visualize metabolic changes due to different stresses. Results indicate promise for visualization of differences in metabolism between malignant and benign tissues and in glandular structures where metabolism is high.

## RESULTS

**Fig. 2.**
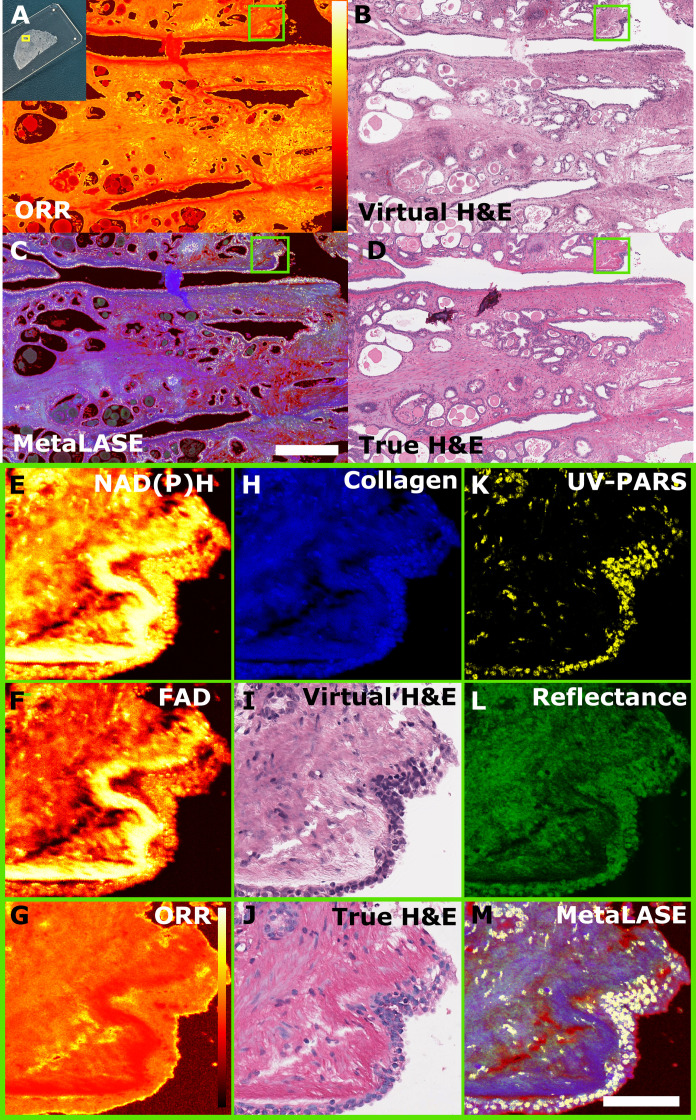
MetaLASE imaging in sectioned FFPE radical prostatectomy specimens. (**A**) ORR metabolic image, (**B**) virtual histology image, (**C**) combined MetaLASE image, and (**D**) corresponding H&E-stained bright-field histology image. Scale bar, 500 μm. Colors in the MetaLASE image correspond to the following: blue, collagen; green, UV scattering; red, ORR; yellow, PARS. From the green square inset in (A) to (D): (**E**, **F**, **H**, **K**, **L**) different captured channel images with NAD(P)H, FAD, collagen, PARS, and UV scattering being shown, respectively; (**G**) calculated ORR image from (E) and (F); (**I**) virtual histology image obtained from (K) and (L); (**J**) corresponding H&E-stained bright-field image; (**M**) MetaLASE image obtained from (G) and (H) and (K) and (L). ORR color bars correspond to a linear scale between 0 and 1. Scale bar, 100 μm.

To demonstrate both metabolic and virtual histology imaging, we used MetaLASE microscopy to image FFPE thin tissue sections, where histological validation was performed post-MetaLASE imaging using H&E-stained bright-field microscopy. Example prostate images of the ORR, virtual histology, MetaLASE blended contrast, and the corresponding true H&E bright-field image are shown in Fig. 2 (A to D), respectively. Here, the MetaLASE blended contrast was formed through the combination of UV-PARS, UV reflectance, collagen autofluorescence, and ORR images using yellow, green, blue, and red colormaps, respectively. The virtual H&E histology images were formed by inputting UV-PARS and UV reflectance data into a trained CycleGAN as previously outlined by Martell *et al.* (*35*). In both Fig. 2 (B and D), pathologists were able to accurately identify features of interest, including regions of benign fibromuscular and glandular tissues. A more rigorous assessment of agreement between true and virtual histology through pathologist studies and quantitative metric analysis was performed by Martell *et al.* (*35*). Furthermore, fig. S8 includes an additional comparison between true H&E-stained bright-field histology and a virtual histology image for a sectioned FFPE radical prostatectomy specimen. Zoomed-in inset images outlined by the green boxes are visualized in Fig. 2 (E to M). The respective NAD(P)H, FAD, ORR, and collagen autofluorescence images are shown in Fig. 2 (E to H), respectively, while UV-PARS and UV reflectance images are shown in Fig. 2 (K and L). The inset region corresponds to a section of a larger draining duct attached to other glands. Comparing the virtual to true H&E histology inset images, strong agreement can be observed between the two images. We are able to observe higher ORR values in glandular regions. Beyond virtual H&E and metabolic contrast, the collagen autofluorescence that is also simultaneously acquired could serve as a label-free alternative to collagen-specific stains such as certain trichrome stains.

**Fig. 3.**
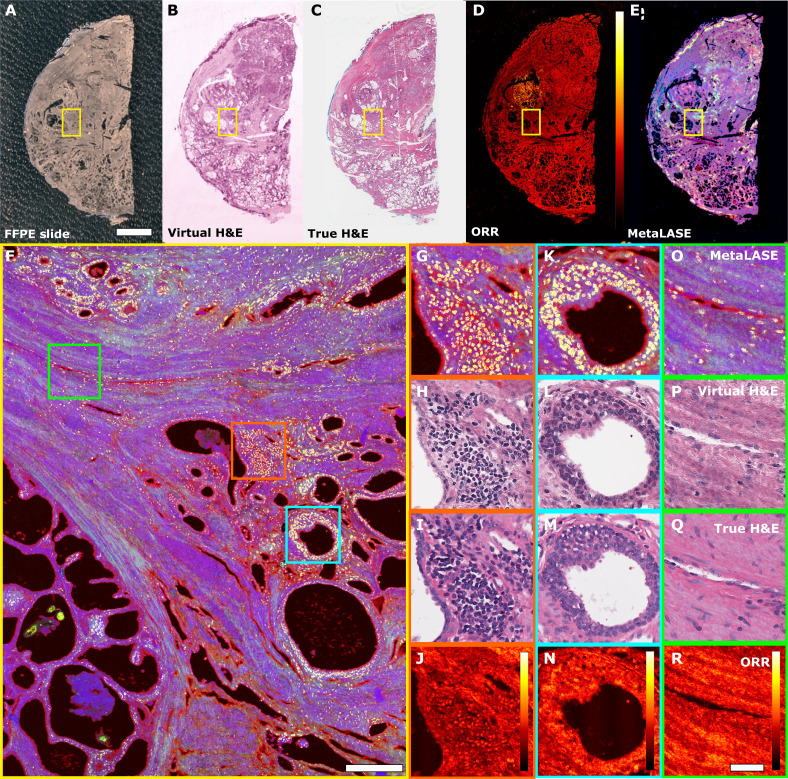
Whole slide MetaLASE imaging in sectioned FFPE radical prostatectomy specimens. (**A** to **E**) Whole slide images showing a photograph of the unstained section, virtual histology image, H&E-stained histology image, ORR image, and MetaLASE image, respectively. The ORR color bar corresponds to a linear scale between 0 and 1. Scale bar, 5 mm. (**F**) MetaLASE image taken from the yellow inset box. Scale bar, 200 μm. Colored square insets show zoom-ins of MetaLASE, corresponding virtual histology, H&E-stained bright-field histology, and calculated ORR images. (**G** to **J**, **K** to **N**, and **O** to **R**) Orange, cyan, and green inset boxes in (F), respectively. ORR color bars correspond to a linear scale between 0 and 1. Scale bar, 50 μm.

To further assess the capabilities of our MetaLASE imaging system, we performed whole slide imaging with Fig. 3 (B, D, and E), showing the virtual histology, ORR, and MetaLASE images, respectively, with a corresponding H&E-stained bright-field image shown in Fig. 3C. Figure 3A shows a photograph of the unstained FFPE-sectioned radical prostatectomy specimen, measuring 3 cm by 1.5 cm, where this slide was imaged in 8 min using asymmetric 0.25-μm by 4-μm stage stepping. We were then able to identify a region of interest, shown here by the yellow box, and rescan using fine stage stepping to obtain a high-resolution MetaLASE image as shown in Fig. 3F. Pathologist assessment of this image has indicated that the top of this image shows the presence of lymphocytes between glands, suggesting chronic inflammation. In Fig. 3D, a region of higher ORR can be seen to border this area of chronic inflammation. In addition, further zoomed-in insets are provided for Fig. 3F, with Fig. 3 (G to J, K to N, and O to R) corresponding to MetaLASE, virtual histology, true H&E-stained histology, and ORR images, respectively. Following pathologist assessment, Fig. 3 (G to J) demonstrates a region of mild chronic inflammation. Figure 3 (K to N) shows a benign gland structure, where the ORR shows higher metabolic activity in the basal layers. This is to be expected because these regions are more actively involved in secretion and regenerative activity. In Fig. 3 (O to R), we can observe a small blood vessel or capillary structure in the fibromuscular tissue. Overall, there is strong agreement between virtual and true H&E-stained histology inset images, with additional ORR contrast providing important information about the metabolic activity of the tissue. Additional examples of MetaLASE imaging in FFPE-sectioned breast and prostate tissue specimens can be found in fig. S9. In fig. S9 (A to C), corresponding to FFPE lumpectomy tissue, pathologists were able to observe a blood vessel in the center of the tissue with surrounding benign fibrous tissue. In addition, in fig. S9 (D to F), corresponding to FFPE prostatectomy tissue, benign fibromuscular tissue with the presence of several compressed glands and chronic inflammation was identified. In fig. S9 (G to I), an additional example of FFPE prostatectomy tissue, invasive carcinoma could be distinguished in virtual histology images, which also correlated with regions of higher ORR intensity.

**Fig. 4.**
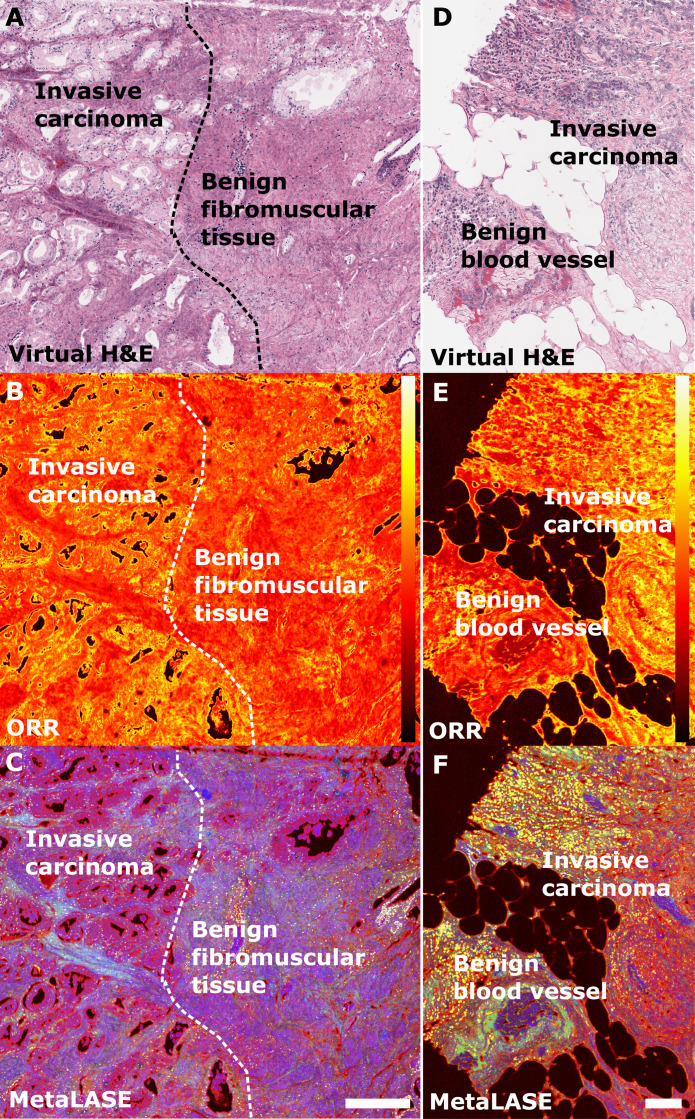
Visualizing regions of higher ORR in radical prostatectomy and breast lumpectomy specimens. (**A** to **C**) Virtual histology, ORR, and MetaLASE images of a radical prostatectomy FFPE thin tissue specimen, respectively. The dotted line is used to divide regions of benign fibromuscular tissue from invasive carcinoma tissue. The ORR color bar corresponds to a linear scale between 0 and 1. Scale bar, 250 μm. (**D** to **F**) Virtual histology, ORR, and MetaLASE images of a lumpectomy FFPE thin tissue specimen, respectively. Scale bar, 125 μm.

To further evaluate the utility of the ORR-based metabolic contrast in our MetaLASE imaging modality, we imaged regions of interest containing both malignant and benign tissues. Examples are shown in both FFPE-sectioned radical prostatectomy and breast lumpectomy tissue specimens. Figure 4A shows a pathologist-annotated virtual histology image of prostatectomy tissue with a clear division between regions of invasive carcinoma and benign fibromuscular tissue. Overlaying these annotations onto the ORR map, as shown in Fig. 4B, it can be seen that regions of invasive carcinoma correspond to higher metabolic activity than regions of benign fibromuscular tissue. Benign glands can also be seen to exhibit elevated metabolic activity as expected. Figure 4D shows a pathologist-annotated virtual histology image of lumpectomy tissue, with clearly defined regions of invasive carcinoma, and the presence of a benign blood vessel. In Fig. 4E, areas of higher metabolic activity align with regions of invasive carcinoma, while high metabolic activity can also be seen in areas of benign inflammation. MetaLASE images shown in Fig. 4 (C and F) are able to showcase these regions of higher ORR in more red areas while also conveying information about collagen content in the more blue areas. Overall, although elevated ORR is not necessarily indicative of neoplasia, these results indicate that there is evidence that invasive carcinoma demonstrates higher values of ORR in comparison to typical benign regions.

**Fig. 5.**
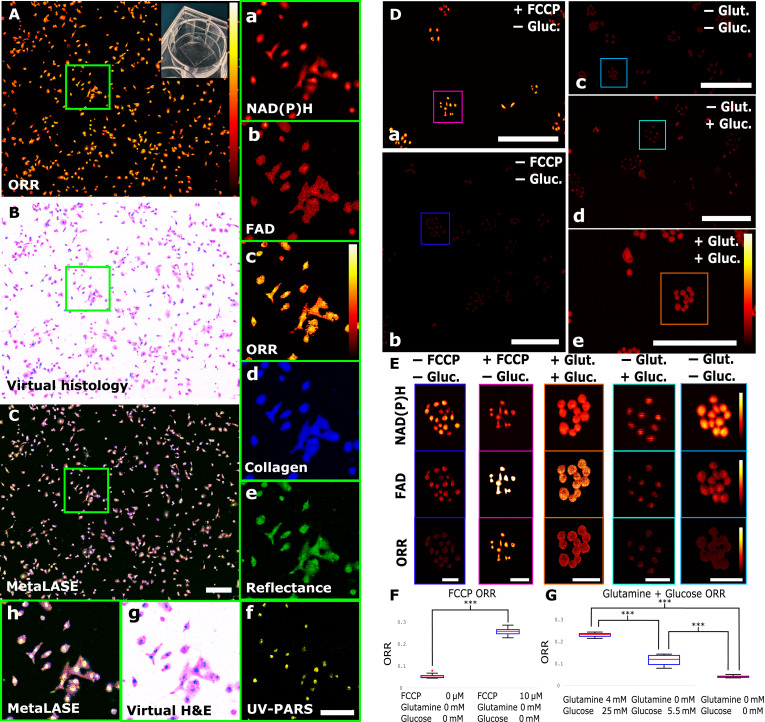
Using MetaLASE to measure metabolic changes in live cells. (**A** to **C**) ORR, virtual histology, and MetaLASE images of live A549 lung carcinoma cells cultured on a glass slide, respectively. Scale bar, 200 μm. (a to h) Green square inset zoom-ins corresponding to NAD(P)H, FAD, calculated ORR, collagen, UV reflectance, UV-PARS, virtual histology, and MetaLASE images, respectively. Scale bar, 50 μm. (**D**) (a to e) Zoomed-in ORR images of cells after specific FCCP/glutamine/glucose addition. Scale bars, 300 μm. (**E**) Zoomed-in NAD(P)H images, FAD images, and ORR inset images corresponding to the colored square insets in (D). ORR color bars correspond to a linear scale between 0 and 1. Scale bars, 100 μm. (**F** and **G**) Box and whisker plots comparing mean ORR values of cells in (D) for FCCP and glutamine/glucose addition, respectively. We indicate *** as *P* < 0.001. Box and whisker plots show the median (central line), upper and lower quartiles (box limits), and 1.5 × interquartile range (whiskers).

Following FFPE-sectioned tissue imaging, we performed live cell experiments involving A549 lung carcinoma cells. Figure 5 (A to C) shows the ORR, virtual histology, and MetaLASE images of these cells, respectively. Because the deep learning–based virtual histology generation introduced by Martell *et al.* (*35*) was trained on representative examples of thin/thick tissue specimen images and not cell culture images, here, we instead adopt a simple pseudocoloring scheme that assigns UV-PARS and UV reflectance images to the red and green channels of a red green, and blue (RGB) array, respectively, while zeroing out the blue channel. Taking the complement of this image, we are able to generate a virtual histology coloration as shown in Fig. 5B. Insets a to h show individual channels corresponding to NAD(P)H, FAD, calculated ORR, collagen, UV reflectance, UV-PARS, virtual histology, and a MetaLASE image, respectively. Here, ORR can be seen to be fairly homogeneous. However, this is to be expected because no stresses have been introduced to the cells. UV-PARS is shown to provide high specificity to nuclear contrast, while UV reflectance and the autofluorescence channels provide complementary information about the cell body.

To assess the ability of our MetaLASE imaging system to measure metabolic changes in live cells, we performed an experiment involving the addition of 10 μM carbonyl cyanide *p*-trifluoromethoxyphenylhydrazone (FCCP) (*36*). Figure 5D (a and b) show zoom-ins of the ORR images obtained following a 10-min 10 μM FCCP incubation period and without FCCP addition, respectively. It can be seen in the selected insets shown in Fig. 5E that, after FCCP is added, there was an observed decrease in the mean NAD(P)H intensity and an increase in the mean FAD intensity, thus resulting in an overall increased mean ORR value. Box and whisker plots characterizing the distribution of mean ORR across a sample of *n* = 149 (total across both cases) cells are shown in Fig. 5F, where the ORR can be seen to have increased by 286% following FCCP addition. Using a two-sample Welch’s *t* test with unequal variances and normal distribution of the data confirmed with the Anderson-Darling test, we find that the mean ORR of each treatment was significantly different (*P* < 0.001). Next, we carried out an experiment involving the addition of glutamine, a nonessential amino acid with elevated involvement in tumor metabolism relative to normal non-neoplastic tissues (*37*). In this experiment, we compared the metabolic change for three cases: 25 mM glutamine and 25 mM glucose addition, no glutamine and 5.5 mM glucose addition, and 24-hour starved with no glutamine or glucose addition. As in the previous experiment, selected zoom-ins of the ORR images obtained for each case are shown in Fig. 5D (c to e), and insets are shown in Fig. 5E. In this experiment, the 24-hour starved cells had a large increase in mean NAD(P)H intensity and decrease in mean FAD intensity, while the fed cells were observed to have a significantly lower mean NAD(P)H intensity and higher mean FAD intensity. As a result, the mean ORR value was observed to increase with glutamine and glucose addition as shown in a box and whisker plot in Fig. 5G. Similarly to the FCCP experiment, we find that the mean ORR of each pair of samples was significantly different (*P* < 0.001), with a sample size of *n* = 248 (total across all three cases).

**Fig. 6.**
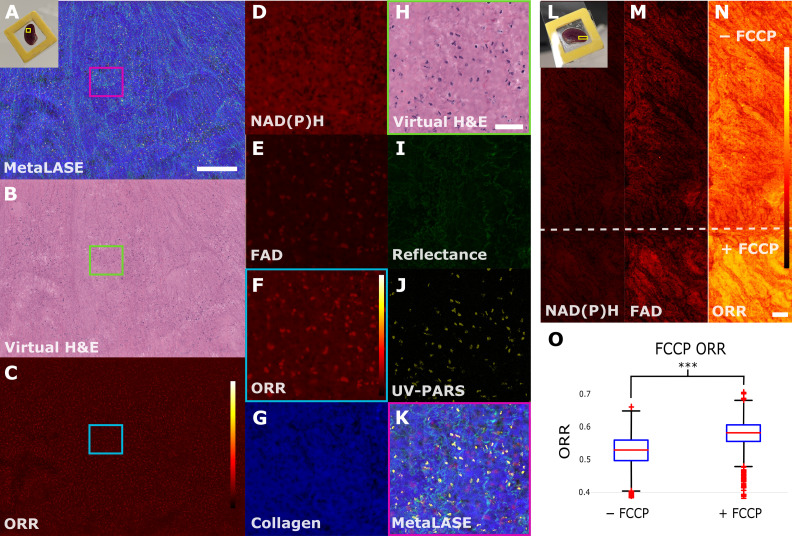
Using MetaLASE to measure metabolic changes in freshly resected murine tissues. (**A** to **C**) MetaLASE, virtual histology, and ORR images of freshly excised murine kidney tissue compressed by a UV transparent coverslip, respectively. Scale bar, 500 μm. (**D** to **K**) Zoomed-in insets of the colored square insets in (A) to (C), with NAD(P)H, FAD, ORR (cyan inset), collagen, virtual histology, UV reflectance, UV-PARS (green inset), and MetaLASE (magenta inset) images being shown, respectively. Scale bar, 50 μm. (**L** to **N**) NAD(P)H, FAD, and ORR images, respectively, of freshly excised murine kidney tissue, where the bottom of the tissue has been submerged in a 10 μM FCCP solution. ORR color bars correspond to a linear scale between 0 and 1. Scale bar, 250 μm. (**O**) Box and whisker plots comparing mean ORR values of tissue in (N) for patches above and below the dashed line. We indicate *** as *P* < 0.001. Box and whisker plots show the median (central line), upper and lower quartiles (box limits), and 1.5 × interquartile range (whiskers). The dashed line has been added to approximate the region of the tissue that was submerged in the FCCP solution.

To demonstrate the ability of our MetaLASE imaging system to image unsectioned fresh tissues, we initially imaged a freshly excised murine kidney mounted between a microscope slide, 3D-printed spacer, and UV transparent coverslip. To ensure tissue viability during transit, freshly excised tissues were preserved in MACS tissue storage solution prior to MetaLASE imaging. The murine kidney was mounted such that the external face was imaged. Figure 6 (A to C) shows the MetaLASE, virtual histology, and ORR images, respectively. In addition, zoomed-in insets d to k show individual channels corresponding to NAD(P)H, FAD, calculated ORR, collagen, virtual histology, and the MetaLASE image, respectively. In these images, it is clear that each channel provides distinct complementary information. UV-PARS is shown to provide high specificity to nuclear contrast, while UV reflectance and the autofluorescence channels provide complementary information about the tissue structure. The ORR also appears to show higher metabolic activity hotspots throughout the tissue.

To assess the feasibility of our MetaLASE approach in assessing metabolic changes in fresh tissues, we again used FCCP, where the bottom edge of a freshly excised murine kidney tissue specimen was submerged in a 10 μM FCCP solution for a 10-min period. The specimen was then washed and imaged along the induced gradient of FCCP as shown in Fig. 6 (L to N). A dashed line has been added to indicate the region of the tissue that was submerged in the FCCP solution. From Fig. 6N, a gradient in the ORR intensity can be observed when across this dashed line. Patches (*n* = 4446, 49 μm by 49 μm) were taken from regions on either side of the dashed line, and the mean ORR value was calculated for each patch. The results were compiled into a box and whisker plot as shown in Fig. 6O, where we observed an increase in the mean ORR value of 11.2% where the FCCP was added to the tissue. By using a two-sample Welch’s *t* test with unequal variances, we find that the mean ORR of each region was significantly different (*P* < 0.001). These results are encouraging as they demonstrate the ability of the MetaLASE system to detect metabolic changes in freshly resected tissue specimens while also being able to simultaneously generate a virtual histology output.

**Fig. 7.**
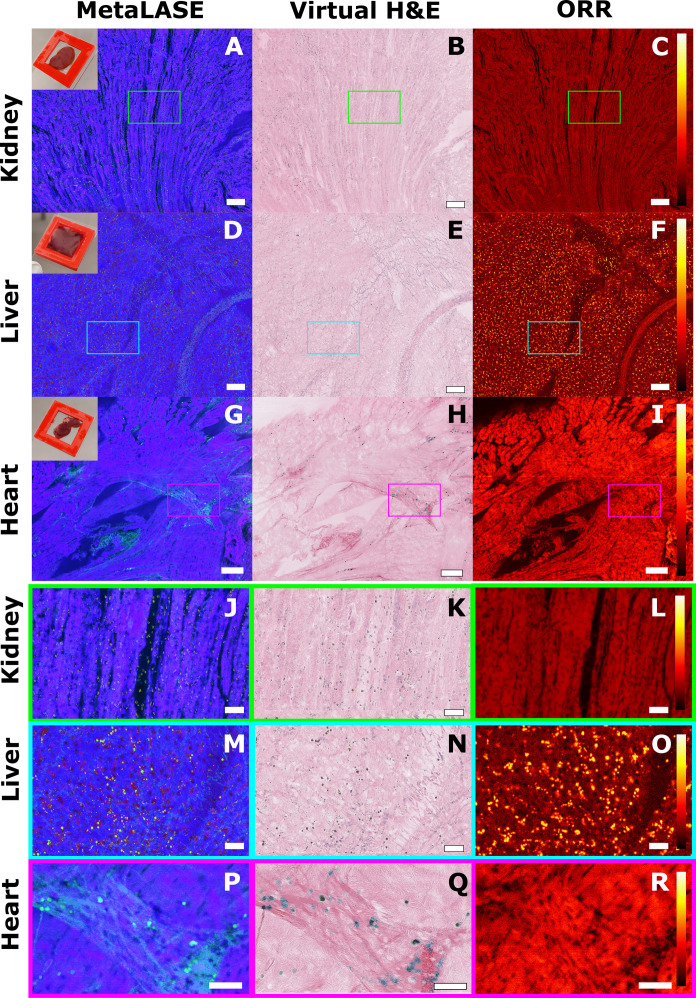
MetaLASE imaging in a variety of freshly resected murine tissues. (**A** to **C**) Ventral axis face of resected kidney tissue taken near the renal pelvis. (**D** to **F**) We observe the outer facet of liver tissue. (**G** to **I**) Outer layer of heart tissue showing both longitudinally and cross-sectional cardiomyocytes. Scale bars, 200 μm. Within each tissue type, we can observe a small inset highlighting various structures. (**J** to **L**) Inset from kidney tissue from the green inset box. (**M** to **O**) Inset from liver tissue from the cyan inset box. (**P** to **R**) Inset from heart tissue from the magenta inset box. ORR color bars correspond to a linear scale between 0 and 1. Scale bars, 50 μm.

To further demonstrate the potential of our MetaLASE imaging system in imaging fresh tissues, we imaged a variety of freshly resected murine tissues. Figure 7 (A to C) shows another example of a murine kidney. However, in comparison to Fig. 6 (A to C), in this instance, the kidney was cut in half along a coronal plane so that the renal medulla could be viewed. In consultation with pathologists, adjacent tubules could be clearly visualized funneling together toward the renal pelvis. Figure 7 (D to F) shows an example of imaging a murine liver. In this case, a lobe of the liver was cut away with the surface being imaged. Pathologists were able to identify the reticulin, along with a large fibrous band corresponding to the capsule. Figure 7 (G to I) shows an example of imaging a murine heart that has been cut in half along a sagittal plane. Pathologists were able to clearly identify cardiomyocytes, arranged more longitudinally in the middle, and in a more cross-sectional arrangement at the top of the field of view. In addition, the voids near the bottom of the image were also identified as a ventricle in the heart. Accompanying insets are provided in Fig. 7 (J to R).

## DISCUSSION

MetaLASE demonstrates the unique ability to generate rich information set above and beyond that provided in conventional histology and to visualize energetics of tissues, which may, in turn, offer clues as to aggressive phenotypes, response to therapies, and more. The fine resolution afforded by high–numerical aperture (NA) focusing of 266-nm UV light enables subcellular details to be resolved, including visualizing cell nuclei, fine tissue architecture, and hotspots in the ORR within the cytoplasm, likely attributed to mitochondrial activity. In addition, MetaLASE offers imaging scales from organelles to tissues, in a variety of specimen types as shown in Figs. 2, 3, 4, 6, and 7. Realistic virtual histology is achieved with close concordance to true H&E histology, as validated by pathologists, while also providing a complimentary metabolic map differentiating metabolically active and malignant tissues from surrounding tissues. In FFPE-sectioned specimens, we are able to observe various local increases in the ORR in known metabolically active structures such as glands or blood vessels in prostate and breast samples. Although results achieved in FFPE-sectioned specimens lack tissue viability and will have a different metabolic profile than live tissues (*38*), informative spatial variations in the ORR have also been observed by others, providing precedence to our imaging studies in fixed tissues (*39*–*41*). MetaLASE is able to accomplish 1-cm^2^ imaging with 250-nm pixel spacing in 7 min, rivaling or exceeding other microscopy platforms and even slide-scanner systems as highlighted in Table 1. In addition, simultaneous acquisition ensures that there can be no spatial or temporal registration errors between both metabolic and virtual histology images caused by potential tissue deformation and/or viability changes between imaging sessions.

Imaging of fresh thick tissues is of great importance to reduce workflow time, preserve tissue architecture, and reduce artifacts. FFPE tissue processing can lead to a variety of imaging artifacts caused by tissue dehydration and cutting steps, damage to DNA/RNA, and reduction in antigenicity (*42*–*44*). In addition, tissue staining can also result in further imaging artifacts and variability (*45*, *46*). By imaging freshly resected thick tissues directly, MetaLASE is able to circumvent these steps, eliminating these potential issues. Imaging in a variety of freshly resected tissue types is demonstrated including the murine kidney, liver, and heart as shown in Fig. 7. Overall, pathologists were able to readily identify many critical features of interest showing the strong versatility and utility of our MetaLASE imaging system.

Our experimental results demonstrating NAD(P)H and FAD autofluorescence changes due to glutamine metabolism could be of considerable interest in future work for discriminating aggressively malignant tumor tissue as glutamine is a nutrient metabolized distinctly in tumors compared to normal tissues (*37*). The results from our experiments summarized in Fig. 5 exhibit elevated metabolic activity under glutamine-fed and glucose-starved conditions, indicating catabolic activity in the A549 cells under conditions of excess glutamine (*47*–*50*). This result is interesting as it demonstrates the potential of MetaLASE microscopy to image tumor-specific changes in metabolic activity. Identifying this hallmark of cancer combined with coregistered virtual histological contrast may aid in differentiating healthy and malignant tissue specimens compared to gold-standard histology alone.

Another method of validating ORR changes in both live cells and freshly resected thick tissues is to intentionally interrupt the electron transport chain using FCCP, a molecule that transports dissociable protons across membranes. We observed a large increase in the ORR of A549 the cells after the addition of FCCP, demonstrating the system’s ability to measure a dynamic change in a living system as seen in Figs. 5 (D to G) and 6 (L to O). We extended this by observing changes in the ORR in fresh tissue with FCCP and observed localized areas of higher ORR corresponding to the presence of FCCP in that section of the tissue. FCCP uncouples adenosine 5′-triphosphate synthesis from the electron transport chain by disrupting the proton gradient across the mitochondrial inner membrane. This drives mitochondrial consumption of reduced form of NAD^+^ (NADH) and FADH_2_ and conversion to the oxidized forms, nicotinamide adenine dinucleotide (NAD^+^) (nonfluorescent) and FAD, respectively. Consequently, the ORR increases as NADH is depleted and additional FAD is formed. These results are corroborated by other studies that have shown similar results (*36*, *43*, *45*, *51*, *52*).

One concern with using ionizing radiation such as UV-C light is phototoxicity, which may lead to cell damage and ultimately cell death. As a result, our MetaLASE approach may not be well suited for applications requiring longitudinal imaging with high cell viability. It is important to note that these considerations are not necessarily applicable with ex vivo freshly excised tissue specimens, where we would expect resected tissues to be formalin-preserved following frontline point-of-care histological and metabolic assessment of tissue margins with our MetaLASE imaging system. Cell viability is quantified and presented in fig. S7. In summary, a 5.6% increase in cell death is observed following a single scan with 350-pJ pulse energy. Pulse energy at the nanojoule-scale is sometimes required for a maximum signal-to-noise ratio in UV-PARS images, although 400-pJ UV-PARS imaging is done routinely in a recent work (*53*). Nevertheless, despite cell viability limitations of using UV-C light for imaging, MetaLASE may still have an important role in quantifying snapshots of metabolism along with simultaneous virtual histology in unstained tissues. Future work should focus on minimizing pulse energy to enable MetaLASE to play a role in possible longitudinal metabolism studies in vitro and in vivo.

Further analysis is required to see if correlations between cell lines with a higher metabolism and specific phenotypes can be observed. Future work should also explore the utilization of machine learning algorithms to predict specific phenotypes from MetaLASE virtual histology and metabolic data, similar to another study (*54*). With the simultaneous acquisition of a multitude of complementary data channels, each with their own unique information, we anticipate many potential avenues where this information could prove invaluable. Currently, predicting cancer aggression usually requires time-consuming and labor-intensive genetic testing, and MetaLASE may offer earlier predictors of aggressive malignant transformation, early detection of therapy-resistant cancers, and enable point-of-care pathology.

Future studies for MetaLASE imaging could include investigating fresh biopsy specimens to produce virtual histology–like images and ORR maps at the point of care, in addition to imaging metabolic dynamics (*55*–*57*) such as response to therapies (*58*) and immune-cell activation (*59*).

## MATERIALS AND METHODS

### Optical imaging system

The MetaLASE microscopy system combines several modular subsystems to acquire multicontrast data using a single UV excitation source. In the excitation path, 266-nm light is generated via second harmonic generation of a 532-nm nanosecond-pulsed source (SPFL-532-40, MKS) through a 4-mm by 4-mm by 10-mm cesium lithium borate crystal (Eksma Optics). The residual 532-nm beam is spectrally separated from the 266-nm beam using a prism (PS863, Thorlabs) and is subsequently collected by a beam dump. The UV beam is then magnified through a variable beam expander (87-565, Edmund Optics) for achieving both collimation and filling the entrance pupil of the system objective. The polarization of the 266-nm beam is then rotated using a half-wave plate (WPH05M-266, Thorlabs) and directed through a polarizing beam splitter (PBS) (10SC16PC.22, Newport), quarter-wave plate (WPQ05M-266, Thorlabs), and a 355-nm longpass dichroic mirror (DM) (Di01-R355-25x36, Semrock) for beam combination with the 1064-nm interrogation beam. Upon back-scattering, the UV beam will have an orthogonal polarization relative to the incident beam and will thus be reflected by the polarizing beam splitter toward a 150-MHz amplified photodiode (PDA10A, Thorlabs) for the 266-nm scattering contrast.

In the absorption detection path, the 1064-nm near-infrared (NIR) beam from a superluminescent diode (SLD-1064-20-YY-350, Innolume) is fiber coupled into a circulator (HPBCIR-1060-H6-L-10-FA-SS, OF-Link) and passed through a zoom collimator (ZC618APC-C, Thorlabs), followed by a 900-nm longpass DM (DMLP900R, Thorlabs) and 355-nm longpass DM (Di01-R355-25x36, Semrock) before being cofocused with the 266-nm beam onto the sample through a 0.5-NA reflective objective (LMM40X-UVV, Thorlabs). Back-scattered NIR light is then redirected via the circulator onto a 75-MHz balanced photodetector (PDB420C-AC, Thorlabs), providing absorption (PARS) contrast.

After sample irradiance, the autofluorescence emission is collected by the system objective and then transmitted through both the 355- and 900-nm longpass DMs and coupled into a multimode fiber (FG105UCA, Thorlabs). The fiber output is collimated (RC08FC-P01, Thorlabs) and directed into an enclosed subsystem for splitting of the tissue autofluorescence into distinct spectral bands corresponding to emission from the collagen (352 to 405 nm), NAD(P)H (439 to 475 nm), and FAD (502 to 548 nm). The fluorescence emission is separated using a series of band-pass filters and DMs corresponding to the collagen channel (84-093, 377 nm, Edmund Optics) (FF414-Di01-25x36, Semrock), the NAD(P)H channel, (86-351, 452 nm, Edmund Optics) (DMLP490R, Thorlabs), and the FAD channel (86-984, 525 nm, Edmund Optics) (Di02-R635-25x36, Semrock). The spectral bands for each channel are then directed onto separate photomultiplier tubes (PMTs). PMTs were used for collagen (H16722P-40, Hamamatsu) and NAD(P)H and FAD (H7422A-40, Hamamatsu Photonics). A visualization of the relevant molecular absorption and emission spectra, as well as our excitation and interrogation wavelengths, is shown in fig. S1.

### Mechanical scanning system

The scanning methodology in this work uses voice-coil stage scanning as demonstrated by Cikaluk *et al.* (*60*). In brief, a linear drive voice-coil stage (X-DMQ12L-AE55D12, Zaber) rapidly oscillates a distance of 2 to 10 mm along one transverse axis while a slow-axis stage (XMS-50S, Newport) traverses the orthogonal direction at a constant velocity, establishing a sinusoidal scanning trajectory over the sample surface. The length of the image is determined by the length of the slow axis stage scan. To trigger the laser, a function generator (DG1022Z, Rigol) generates pulses at a repetition rate ranging from 10 to 2000 kHz, with the pulse repetition rate being chosen to obtain the desired spatial resolution for an oscillation frequency and scanning distance. The generated pulses are then used to trigger the laser and a digital delay generator (DG645, SRS) for external triggering of the data acquisition (DAQ) card for excitation event recognition and for resetting of the system’s analog peak detectors used in signal acquisition.

### Data acquisition

In this work, eight data channels were simultaneously sampled every 20 ns during imaging. For a precise reconstruction of the voice-coil position trajectory, the quadrature encoder channels from the stage must be recorded continuously over the duration of the scan. As such, a 100-MHz 14-bit digitizer card (CSE8389, GaGe Applied) was used to stream all data channels at 50 megasamples/second (MS/s). The DAQ channels include two quadrature encoder channels for absolute position reconstruction, a digital trigger signal for excitation event recognition, the UV-PARS channel, the UV reflectance channel, and three autofluorescence channels [collagen, NAD(P)H, and FAD]. The streaming rate of 50 MS/s was determined to be the maximum practical streaming rate for eight channel continuous streaming with the given acquisition system. Because the 20-ns sampling interval is too coarse to capture the rapidly developed optical signals, each signal is conditioned prior to digitization. Absorption contrast data points were extracted from the digitized waveform by integrating the PARS signal over a modulation window. To measure the magnitude of the back-scattered 266-nm pulses, a custom-made peak detector (*61*) was used at the output of the UV reflectance channel photodiode prior to digitization. For the FAD and NAD(P)H autofluorescence channels, the output of the PMT (H7422PA-40, Hamamatsu) is amplified using a 300-MHz transimpedance amplifier (C11184, Hamamatsu), inverted using a 340-MHz operational amplifier (THS3001EVM, Texas Instruments), and subsequently passed through a sample-and-hold peak detector circuit prior to digitization. For the collagen channel, the output of the PMT (H16722P-40, Hamamatsu) used the same amplification, inverting and peak detection as described above. See fig. S2 for a full system diagram and fig. S3 for the acquisition timing diagram for each channel and the signal amplification process.

### Image reconstruction

To render data acquired from the digitizer channels as an image, the scan trajectory was reconstructed from AquadB encoder data using the custom C++ OpenMP multithreaded software for parallel read-in of encoder and optical signals, resolving stage positions from encoder state changes (fast axis) and sample timestamps (slow axis) and associating optical signal data with each position on the sinusoidal trajectory. Using Delaunay triangulation–based scattered data interpolation in a central processing unit-parallelized MATLAB script. This scan data was rendered onto a pixelated Cartesian grid using natural neighbor interpolation to form image arrays for each data channel. Total image formation required ~2.7 min/mm^2^ using a 16-thread processor (i9-9900k, Intel) with 128 GB of RAM (random-access memory), with considerable opportunity for speed-up of the interpolation rendering component using graphics processing unit-based acceleration, and the scan trajectory reconstruction component using hardware-based (e.g., field programmable gate arrays) implementations.

To generate virtual histology images, the PARS and UV reflectance channels were used as virtual H&E channels, respectively. To additionally achieve maximally realistic stain style, we used a CycleGAN, discussed in detail by Martell *et al.* (*35*). In brief, PARS and UV reflectance channels were assigned to red and green color channels in an RGB array, with the blue color channel remaining empty. Image color inversion was then performed to produce a background matching that of bright-field histology for network training. Over 15,000 unpaired pseudo-color virtual histology images and true H&E histology images were subsequently used as inputs to a CycleGAN algorithm for training. Once trained, pseudo-color inputs were transformed by the algorithm into maximally realistic virtual H&E images.

To generate a MetaLASE image, various optical contrast channels were combined into an 8-bit RGB image. The red, green, and blue color channels were assigned to the ORR, UV scattering, and collagen autofluorescence, respectively. In addition, intrinsically sparse nucleic contrast was superimposed on the RGB image in a yellow-white colormap. Image processing to then obtain the image was applied. Outlier pixels above the 98th percentile of the max image intensity were removed for each channel.

### Autofluorescence system response characterization

We characterized the linearity of the NAD(P)H and FAD autofluorescence PMT response. We did so using a dilution curve of NAD(P)H and FAD and compared the known concentrations to the measured voltage from the PMT, amplifier, and subsequent DAQ card readings. NAD(P)H and FAD concentrations ranging between 1 and 500 μM were used. The PMT voltage signal as a function of dilution concentration data was fit linearly with *R*^2^ values of 0.891 and 0.821 for NAD(P)H and FAD, respectively, similar to a previous work (*45*). See fig. S4 for the linearity plots. In addition, because autofluorescence measurements were obtained through a peak detection circuit, the linearity of this peak detector was also measured. To do this, two measurements were simultaneously captured using a T connection. The first was the output from the PMT onto a 500-MHz oscilloscope (DPO 7054, Tektronix), and the other was the output from the peak detector. By varying the incident pulse energy on the sample, data were collected for different PMT output voltages. Results are shown in fig. S5 where the data have been fit linearly with an *R*^2^ value of 0.965.

### Resolution characterization

Phantom imaging was performed with all MetaLASE channels. An error function edge spread function was fit across the intensity plot for each channel. The derivative of this fit was calculated to obtain a point spread function (PSF) whose full width at half maximum (FWHM) was measured. Lateral resolution was defined as the FWHM of the PSF. The lateral spatial resolutions of the various MetaLASE channels were characterized by the following measurements: 0.945 μm for collagen autofluorescence, 1.045 μm for NAD(P)H autofluorescence, 1.134 μm for FAD autofluorescence, 0.400 μm for UV scattering, and 0.273 μm for UV-PARS, as seen in fig. S6.

### Tissue acquisition and preparation

Formalin-fixed bread-loafed human lumpectomy and radical prostatectomy tissue specimens were obtained from patients with breast and prostate cancer after pathology cases were closed and tissues were flagged for disposal as per approved ethics [HREBA (Cancer)/HREBA.CC-20-0145]. Benign breast tissue specimens were obtained from a reduction mammoplasty procedure, where the tissue would have otherwise been discarded. Patient details pertaining to excised tissue specimens were kept anonymous from research staff and pathologists. Tissue specimens used in imaging were first paraffin embedded, then sectioned into 4-μm thin sections, followed by deparaffination and rehydration prior to imaging. Deparaffination of tissue sections was carried out by heating the tissue slides at 60°C for 1 hour, followed by 5-min washes in two changes of xylene, two changes of 100% ethanol, 95% ethanol, and deionized (DI) water.

### Cell culture preparation and glutamine protocol

A549 cells were obtained (CCL-185, American Type Culture Collection), and cells were confirmed to be free from mycoplasma. Cells were cultured at 37°C in 9% CO_2_ in Dulbecco’s modified Eagle’s medium (DMEM) [Gibco, no. 11995-073; glucose (25 mM) and glutamine (4 mM)] supplemented with 10% fetal bovine serum (Sigma-Aldrich, no. F1051) and 1% PenStrep (Gibco, no. 15240062). For glutamine starvation experiments, cells were grown up to 60% confluency in complete media, washed once with phosphate-buffered saline (PBS) then cultured in complete media or serum-free and glutamine-free DMEM media (Gibco, no. A1443001) and 4 mM glucose for 24 and 48 hours on glass slides prior to imaging.

### FCCP protocol

A549 cells were removed from their culture media and washed with PBS. Cells were then left to incubate at room temperature with 10 μM FCCP (Sigma-Aldrich, no. C2920) for 10 min before being washed with PBS once and then DI water once before the cultured cell plate was transferred onto a microscope slide in preparation for imaging. For thick tissue experiments, freshly resected tissue was washed with PBS once, then partially submerged in 10 μM FCCP for 10 min, rewashed with PBS once followed by DI water once, and then inserted into our imaging sample holder prior to imaging.

### Cell viability testing

Cell viability was assessed via propidium iodide (PI) staining, which selectively stains the nuclei of dead cells that have compromised cell membranes. Cells were stained in 500 nM PI in PBS after imaging and incubated for 10 min at room temperature while still adhered to the coverslip. Cells were then washed with two changes of PBS to remove unbound PI. Cells were then fixed by incubation in 4% formaldehyde. Cells were counterstained with 300 nM 4′,6-diamidino-2-phenylindole (DAPI) for nuclear contrast (including previously live cells) and Alexa Fluor 488 wheat germ agglutinin (1 mg/ml; AF488-WGA) membrane stain and incubated for 10 min at room temperature. Coverslips were briefly submerged in DI water to remove salts and unbound stains. Coverslips were then mounted onto slides using the ProLong Gold Antifade Mountant. Whole slide imaging was performed on a ZEISS AxioScan slide scanner at 20x. Analysis was performed on the ZEISS ZEN software. Imaging segmentation was performed to create masks for individual cells using the AF488-WGA membrane staining. Thresholds above the autofluorescence background were set for DAPI and PI stains. Dead cells due to laser exposure were determined by the number of cell masks containing a sufficient PI signal, and the total number of cells was identified by the number of cell masks containing DAPI staining. Live cells were the difference between total and dead cells.

### Statistical analysis and reproducibility

To statistically assess the information provided by ORR imaging under varied treatment conditions, *P* values were determined using a two-sample, two-tailed Welch’s unequal variances *t* test. This tests the null hypothesis of the two populations having the same mean ORR against the alternative of different mean ORR values for the two treatment groups. Normality of the data was verified using an Anderson-Darling test. No statistical method was used to predetermine sample sizes. Corrections for multiple comparisons were not performed. All images depict single scans of single samples, although all data sets represent multiple repeated imaging experiments where structural and metabolic features were routinely resolved.

## Supplementary Materials

### This PDF file includes:

Supplementary Text Figs. S1 to S9 References

## References

[1] V. Di Gialleonardo, D. M. Wilson, K. R. Keshari, The potential of metabolic imaging. Semin. Nucl. Med. 46, 28–39 (2016). 26687855 10.1053/j.semnuclmed.2015.09.004 PMC4686865

[2] O. I. Kolenc, K. P. Quinn, Evaluating cell metabolism through autofluorescence imaging of NAD(P)H and FAD. Antioxid. Redox Signal. 30, 875–889 (2019). 29268621 10.1089/ars.2017.7451 PMC6352511

[3] I. Georgakoudi, K. P. Quinn, Label-free optical metabolic imaging in cells and tissues. Annu. Rev. Biomed. Eng. 25, 413–443 (2023). 37104650 10.1146/annurev-bioeng-071516-044730 PMC10733979

[4] B. Chance, B. Schoener, R. Oshino, F. Itshak, Y. Nakase, Oxidation-reduction ratio studies of mitochondria in freeze-trapped samples. NADH and flavoprotein fluorescence signals. J. Biol. Chem. 254, 4764–4771 (1979). 220260

[5] A. Shiino, M. Haida, B. Beauvoit, B. Chance, Three-dimensional redox image of the normal gerbil brain. Neuroscience 91, 1581–1585 (1999). 10391462 10.1016/s0306-4522(98)00670-8

[6] L. E. Navas, A. Carnero, NAD^+^ metabolism, stemness, the immune response, and cancer. Signal Transduct. Target. Ther. 6, 2 (2021). 33384409 10.1038/s41392-020-00354-w PMC7775471

[7] M. A. Yaseen, S. Sakadžić, W. Wu, W. Becker, K. A. Kasischke, D. A. Boas, In vivo imaging of cerebral energy metabolism with two-photon fluorescence lifetime microscopy of NADH. Biomed. Opt. Express 4, 307–321 (2013). 23412419 10.1364/BOE.4.000307 PMC3567717

[8] S. Palmer, K. Litvinova, E. U. Rafailov, G. Nabi, Detection of urinary bladder cancer cells using redox ratio and double excitation wavelengths autofluorescence. Biomed. Opt. Express 6, 977–986 (2015). 25798319 10.1364/BOE.6.000977 PMC4361449

[9] H. N. Xu, S. Nioka, J. D. Glickson, B. Chance, L. Z. Li, Quantitative mitochondrial redox imaging of breast cancer metastatic potential. J. Biomed. Opt. 15, 036010 (2010). 20615012 10.1117/1.3431714 PMC3188620

[10] H. N. Xu, J. Tchou, L. Z. Li, Redox imaging of human breast cancer core biopsies: A preliminary investigation. Acad. Radiol. 20, 764–768 (2013). 23664401 10.1016/j.acra.2013.02.006 PMC3791620

[11] J. Xu, X. Luo, G. Wang, H. Gilmore, A. Madabhushi, A deep convolutional neural network for segmenting and classifying epithelial and stromal regions in histopathological images. Neurocomputing 191, 214–223 (2016). 28154470 10.1016/j.neucom.2016.01.034 PMC5283391

[12] I. Sabdyusheva Litschauer, K. Becker, S. Saghafi, S. Ballke, C. Bollwein, M. Foroughipour, J. Gaugeler, M. Foroughipour, V. Schavelová, V. László, B. Döme, C. Brostjan, W. Weichert, H. U. Dodt, 3D histopathology of human tumours by fast clearing and ultramicroscopy. Sci. Rep. 10, 17619 (2020). 33077794 10.1038/s41598-020-71737-w PMC7572501

[13] L. Waaijer, M. D. Filipe, J. Simons, C. C. van der Pol, T. de Boorder, P. J. van Diest, A. J. Witkamp, Detection of breast cancer precursor lesions by autofluorescence ductoscopy. Breast Cancer 28, 119–129 (2021). 32725533 10.1007/s12282-020-01136-6 PMC7796885

[14] M. C. Skala, K. M. Riching, A. Gendron-Fitzpatrick, J. Eickhoff, K. W. Eliceiri, J. G. White, N. Ramanujam, In vivo multiphoton microscopy of NADH and FAD redox states, fluorescence lifetimes, and cellular morphology in precancerous epithelia. Proc. Natl. Acad. Sci. U.S.A. 104, 19494–19499 (2007). 18042710 10.1073/pnas.0708425104 PMC2148317

[15] B. Lin, S. Urayama, R. M. G. Saroufeem, D. L. Matthews, S. G. Demos, Characterizing the origin of autofluorescence in human esophageal epithelium under ultraviolet excitation. Opt. Express 18, 21074–21082 (2010). 20941003 10.1364/OE.18.021074

[16] R. Cao, H. K. Wallrabe, A. Periasamy, Multiphoton FLIM imaging of NAD(P)H and FAD with one excitation wavelength. J. Biomed. Opt. 25, 014510 (2020). 31920048 10.1117/1.JBO.25.1.014510 PMC6951488

[17] G. Fürtjes, D. Reinecke, N. von Spreckelsen, A.-K. Meißner, D. Rueß, M. Timmer, C. Freudiger, A. Ion-Margineanu, F. Khalid, K. Watrinet, C. Mawrin, A. Chmyrov, R. Goldbrunner, O. Bruns, V. Neuschmelting, Intraoperative microscopic autofluorescence detection and characterization in brain tumors using stimulated Raman histology and two-photon fluorescence. Front. Oncol. 13, 1146031 (2023). 37234975 10.3389/fonc.2023.1146031 PMC10207900

[18] N. J. M. Haven, K. L. Bell, P. Kedarisetti, J. D. Lewis, R. J. Zemp, Ultraviolet photoacoustic remote sensing microscopy. Opt. Lett. 44, 3586–3589 (2019). 31305578 10.1364/OL.44.003586

[19] N. J. Haven, P. Kedarisetti, B. S. Restall, R. J. Zemp, Reflective objective-based ultraviolet photoacoustic remote sensing virtual histopathology. Opt. Lett. 45, 535–538 (2020).

[20] P. Hajireza, W. Shi, K. Bell, R. J. Paproski, R. J. Zemp, Non-interferometric photoacoustic remote sensing microscopy. Light Sci. Appl. 6, e16278–e16278 (2017). 30167263 10.1038/lsa.2016.278 PMC6062239

[21] P. H. Reza, K. Bell, W. Shi, J. Shapiro, R. J. Zemp, Deep non-contact photoacoustic initial pressure imaging. Optica 5, 814–820 (2018).

[22] N. J. Haven, M. T. Martell, H. Li, J. D. Hogan, R. J. Zemp, Investigating mechanisms of laser pulse-induced reflectivity modulations in photoacoustic remote sensing with a 10 million frames-per-second camera. Sci. Rep. 13, 3751 (2023). 36882492 10.1038/s41598-023-30831-5 PMC9992668

[23] B. S. Restall, B. D. Cikaluk, M. T. Martell, N. J. M. Haven, R. Mittal, S. Silverman, L. Peiris, J. Deschenes, B. A. Adam, A. Kinnaird, R. J. Zemp, Fast hybrid optomechanical scanning photoacoustic remote sensing microscopy for virtual histology. Biomed. Opt. Express 13, 39–47 (2022). 35154852 10.1364/BOE.443751 PMC8803023

[24] S. Abbasi, M. Le, B. Sonier, D. Dinakaran, G. Bigras, K. Bell, J. R. Mackey, P. Haji Reza, All-optical reflection-mode microscopic histology of unstained human tissues. Sci. Rep. 9, 13392 (2019). 31527734 10.1038/s41598-019-49849-9 PMC6746717

[25] B. Ecclestone, D. Dinakaran, P. H. Reza, Single acquisition label-free histology-like imaging with dual-contrast photoacoustic remote sensing microscopy. J. Biomed. Opt. 26, 056007 (2021). 34036757 10.1117/1.JBO.26.5.056007 PMC8144614

[26] B. S. Restall, N. J. Haven, P. Kedarisetti, M. T. Martell, B. D. Cikaluk, S. Silverman, L. Peiris, J. Deschenes, R. J. Zemp, Virtual hematoxylin and eosin histopathology using simultaneous photoacoustic remote sensing and scattering microscopy. Opt. Express 29, 13864–13875 (2021). 33985114 10.1364/OE.423740

[27] P. Kedarisetti, B. S. Restall, N. J. Haven, M. T. Martell, B. D. Cikaluk, J. Deschenes, R. J. Zemp, F-mode ultraviolet photoacoustic remote sensing for label-free virtual H&E histopathology using a single excitation wavelength. Opt. Lett. 46, 3500–3503 (2021). 34329209 10.1364/OL.426543

[28] K. Bell, S. Abbasi, D. Dinakaran, M. Taher, G. Bigras, F. K. van Landeghem, J. R. Mackey, P. Haji Reza, Reflection-mode virtual histology using photoacoustic remote sensing microscopy. Sci. Rep. 10, 19121 (2020). 33154496 10.1038/s41598-020-76155-6 PMC7644651

[29] N. Pellegrino, B. R. Ecclestone, D. Dinakaran, F. van Landeghem, P. Fieguth, P. H. Reza, Time-domain feature extraction for target specificity in photoacoustic remote sensing microscopy. Opt. Lett. 47, 3952–3955 (2022). 35913356 10.1364/OL.457142

[30] M. T. Martell, N. J. Haven, R. J. Zemp, Multimodal imaging with spectral-domain optical coherence tomography and photoacoustic remote sensing microscopy. Opt. Lett. 45, 4859–4862 (2020). 32870876 10.1364/OL.398940

[31] B. R. Ecclestone, Z. Hosseinaee, N. Abbasi, K. Bell, D. Dinakaran, J. R. Mackey, P. Haji Reza, Three-dimensional virtual histology in unprocessed resected tissues with photoacoustic remote sensing (PARS) microscopy and optical coherence tomography (OCT). Sci. Rep. 11, 13723 (2021). 34215785 10.1038/s41598-021-93222-8 PMC8253737

[32] M. T. Martell, N. J. Haven, R. J. Zemp, Fiber-based photoacoustic remote sensing microscopy and spectral-domain optical coherence tomography with a dual-function 1050-nm interrogation source. J. Biomed. Opt. 26, 066502 (2021). 34164968 10.1117/1.JBO.26.6.066502 PMC8220968

[33] B. S. Restall, P. Kedarisetti, N. J. Haven, M. T. Martell, R. J. Zemp, Multimodal 3D photoacoustic remote sensing and confocal fluorescence microscopy imaging. J. Biomed. Opt. 26, 096501 (2021). 34523269 10.1117/1.JBO.26.9.096501 PMC8440567

[34] N. J. Haven, M. T. Martell, B. D. Cikaluk, B. S. Restall, E. McAlister, S. Silverman, L. Peiris, J. Deschenes, X. Li, R. J. Zemp, Virtual histopathology with ultraviolet scattering and photoacoustic remote sensing microscopy. Opt. Lett. 46, 5153–5156 (2021). 34653139 10.1364/OL.436136

[35] M. T. Martell, N. J. Haven, B. D. Cikaluk, B. S. Restall, E. A. McAlister, R. Mittal, B. A. Adam, N. Giannakopoulos, L. Peiris, S. Silverman, J. Deschenes, X. Li, R. J. Zemp, Deep learning-enabled realistic virtual histology with ultraviolet photoacoustic remote sensing microscopy. Nat. Commun. 14, 5967 (2023). 37749108 10.1038/s41467-023-41574-2 PMC10519961

[36] M. A. Yaseen, J. Sutin, W. Wu, B. Fu, H. Uhlirova, A. Devor, D. A. Boas, S. Sakadžić, Fluorescence lifetime microscopy of nadh distinguishes alterations in cerebral metabolism in vivo. Biomed. Opt. Express 8, 2368–2385 (2017). 28663879 10.1364/BOE.8.002368 PMC5480486

[37] C. T. Hensley, A. T. Wasti, R. J. DeBerardinis, Glutamine and cancer: Cell biology, physiology, and clinical opportunities. J. Clin. Invest. 123, 3678–3684 (2013). 23999442 10.1172/JCI69600 PMC3754270

[38] A. Sánchez-Hernández, C. M. Polleys, I. Georgakoudi, Formalin fixation and paraffin embedding interfere with preservation of optical metabolic assessments based on endogenous NAD(P)H and FAD two photon excited fluorescence. bioRxiv 2023.06.16.545363 [Preprint] (2023). 10.1101/2023.06.16.545363. PMC10581792 37854574

[39] L. Z. Li, M. Masek, T. Wang, H. N. Xu, S. Nioka, J. A. Baur, T. M. Ragan, Two-photon autofluorescence imaging of fixed tissues: Feasibility and potential values for biomedical applications. Adv. Exp. Med. Biol. 1232, 375–381 (2020). 31893434 10.1007/978-3-030-34461-0_48 PMC7183211

[40] H. N. Xu, H. Zhao, K. Chellappa, J. G. Davis, S. Nioka, J. A. Baur, L. Z. Li, Optical redox imaging of fixed unstained muscle slides reveals useful biological information. Mol. Imaging Biol. 21, 417–425 (2019). 30977079 10.1007/s11307-019-01348-z PMC6581512

[41] C. J. Jenvey, J. R. Stabel, Autofluorescence and nonspecific immunofluorescent labeling in frozen bovine intestinal tissue sections: Solutions for multicolor immunofluorescence experiments. J. Histochem. Cytochem. 65, 531–541 (2017). 28763246 10.1369/0022155417724425 PMC5582670

[42] S. Chatterjee, Artefacts in histopathology. J. Oral. Maxillofac. Pathol. 18, S111–S116 (2014). 25364159 10.4103/0973-029X.141346 PMC4211218

[43] A. Y. Abramov, M. Gegg, A. Grunewald, N. W. Wood, C. Klein, A. H. V. Schapira, Bioenergetic consequences of PINK1 mutations in Parkinson disease. PLOS ONE 6, e25622 (2011). 22043288 10.1371/journal.pone.0025622 PMC3197155

[44] R. Xie, J.-Y. Chung, K. Ylaya, R. L. Williams, N. Guerrero, N. Nakatsuka, C. Badie, S. M. Hewitt, Factors influencing the degradation of archival formalin-fixed paraffin-embedded tissue sections. J. Histochem. Cytochem. 59, 356–365 (2011). 21411807 10.1369/0022155411398488 PMC3201147

[45] J. Hou, H. J. Wright, N. S.-K. Chan, R. D. H. Tran, O. V. Razorenova, E. O. Potma, B. J. Tromberg, Correlating two-photon excited fluorescence imaging of breast cancer cellular redox state with seahorse flux analysis of normalized cellular oxygen consumption. J. Biomed. Opt. 21, 060503 (2016). 27300321 10.1117/1.JBO.21.6.060503 PMC4906146

[46] J. M. Corbin, M. J. Ruiz-Echevarria, One-carbon metabolism in prostate cancer: The role of androgen signaling. Int. J. Mol. Sci. 17, 1208 (2016). 27472325 10.3390/ijms17081208 PMC5000606

[47] L. Zhu, K. Ploessl, R. Zhou, D. Mankoff, H. F. Kung, Metabolic imaging of glutamine in cancer. J. Nucl. Med. 58, 533–537 (2017). 28232608 10.2967/jnumed.116.182345 PMC5373500

[48] J. Jiang, S. Srivastava, J. Zhang, Starve cancer cells of glutamine: Break the spell or make a hungry monster? Cancers 11, 804 (2019). 31212591 10.3390/cancers11060804 PMC6627209

[49] J. Jin, J.-K. Byun, Y.-K. Choi, K.-G. Park, Targeting glutamine metabolism as a therapeutic strategy for cancer. Exp. Mol. Med. 55, 706–715 (2023). 37009798 10.1038/s12276-023-00971-9 PMC10167356

[50] A. Varone, J. Xylas, K. P. Quinn, D. Pouli, G. Sridharan, M. E. McLaughlin-Drubin, C. Alonzo, K. Lee, K. Münger, I. Georgakoudi, Endogenous two-photon fluorescence imaging elucidates metabolic changes related to enhanced glycolysis and glutamine consumption in precancerous epithelial tissues. Cancer Res. 74, 3067–3075 (2014). 24686167 10.1158/0008-5472.CAN-13-2713 PMC4837452

[51] K. M. Holmström, L. Baird, Y. Zhang, I. Hargreaves, A. Chalasani, J. M. Land, L. Stanyer, M. Yamamoto, A. T. Dinkova-Kostova, A. Y. Abramov, Nrf2 impacts cellular bioenergetics by controlling substrate availability for mitochondrial respiration. Biol. Open 2, 761–770 (2013). 23951401 10.1242/bio.20134853 PMC3744067

[52] A. U. Rehman, A. G. Anwer, M. E. Gosnell, S. B. Mahbub, G. Liu, E. M. Goldys, Fluorescence quenching of free and bound NADH in HeLa cells determined by hyperspectral imaging and unmixing of cell autofluorescence. Biomed. Opt. Express 8, 1488–1498 (2017). 28663844 10.1364/BOE.8.001488 PMC5480559

[53] B. R. Ecclestone, K. Bell, S. Sparkes, D. Dinakaran, J. R. Mackey, P. Haji Reza, Label-free complete absorption microscopy using second generation photoacoustic remote sensing. Sci. Rep. 12, 8464 (2022). 35589763 10.1038/s41598-022-11235-3 PMC9120477

[54] J. Jiang, M. Feng, A. Jacob, L. Z. Li, H. N. Xu, *Oxygen Transport to Tissue XLII* (Springer, 2021), pp. 253–258.

[55] A. J. Walsh, R. S. Cook, H. C. Manning, D. J. Hicks, A. Lafontant, C. L. Arteaga, M. C. Skala, Optical metabolic imaging identifies glycolytic levels, subtypes, and early-treatment response in breast cancer. Cancer Res. 73, 6164–6174 (2013). 24130112 10.1158/0008-5472.CAN-13-0527 PMC3801432

[56] A. J. Walsh, J. A. Castellanos, N. S. Nagathihalli, N. B. Merchant, M. C. Skala, Optical imaging of drug-induced metabolism changes in murine and human pancreatic cancer organoids reveals heterogeneous drug response. Pancreas 45, 863 (2016). 26495796 10.1097/MPA.0000000000000543 PMC4874911

[57] Z. Liu, D. Pouli, C. A. Alonzo, A. Varone, S. Karaliota, K. P. Quinn, K. Münger, K. P. Karalis, I. Georgakoudi, Mapping metabolic changes by noninvasive, multiparametric, high-resolution imaging using endogenous contrast. Sci. Adv. 4, eaap9302 (2018). 29536043 10.1126/sciadv.aap9302 PMC5846284

[58] A. J. Walsh, R. S. Cook, M. E. Sanders, L. Aurisicchio, G. Ciliberto, C. L. Arteaga, M. C. Skala, Quantitative optical imaging of primary tumor organoid metabolism predicts drug response in breast cancer. Cancer Res. 74, 5184–5194 (2014). 25100563 10.1158/0008-5472.CAN-14-0663 PMC4167558

[59] A. J. Walsh, K. P. Mueller, K. Tweed, I. Jones, C. M. Walsh, N. J. Piscopo, N. M. Niemi, D. J. Pagliarini, K. Saha, M. C. Skala, Classification of T-cell activation via autofluorescence lifetime imaging. Nat. Biomed. Eng. 5, 77–88 (2021). 32719514 10.1038/s41551-020-0592-z PMC7854821

[60] B. D. Cikaluk, B. S. Restall, N. J. Haven, M. T. Martell, E. A. McAlister, R. J. Zemp, Rapid ultraviolet photoacoustic remote sensing microscopy using voice-coil stage scanning. Opt. Express 31, 10136–10149 (2023). 37157568 10.1364/OE.481313

[61] L. Snider, K. Bell, P. Hajireza, R. J. Zemp, Toward wide-field high-speed photoacoustic remote sensing microscopy, in *Photons Plus Ultrasound: Imaging and Sensing 2018* (SPIE, 2018), vol. 10494, pp. 143–150.

[62] M. Broekgaarden, A.-L. Bulin, J. Frederick, Z. Mai, T. Hasan, Tracking photodynamic-and chemotherapy-induced redox-state perturbations in 3D culture models of pancreatic cancer: A tool for identifying therapy-induced metabolic changes. J. Clin. Med. 8, 1399 (2019). 31500115 10.3390/jcm8091399 PMC6788194

[63] D. Reichert, L. I. Wadiura, M. T. Erkkilae, J. Gesperger, A. Lang, T. Roetzer-Pejrimovsky, J. Makolli, A. Woehrer, M. Wilzbach, C. Hauger, B. Kiesel, M. Andreana, A. Unterhuber, W. Drexler, G. Widhalm, R. A. Leitgeb, Flavin fluorescence lifetime and autofluorescence optical redox ratio for improved visualization and classification of brain tumors. Front. Oncol. 13, 1105648 (2023). 36890834 10.3389/fonc.2023.1105648 PMC9986542

[64] S. You, H. Tu, E. J. Chaney, Y. Sun, Y. Zhao, A. J. Bower, Y.-Z. Liu, M. Marjanovic, S. Sinha, Y. Pu, S. A. Boppart, Intravital imaging by simultaneous label-free autofluorescence-multiharmonic microscopy. Nat. Commun. 9, 2125 (2018). 29844371 10.1038/s41467-018-04470-8 PMC5974075

[65] M. A. Ilie, C. Caruntu, M. Lupu, D. Lixandru, M. Tampa, S.-R. Georgescu, A. Bastian, C. Constantin, M. Neagu, S. A. Zurac, D. Boda, Current and future applications of confocal laser scanning microscopy imaging in skin oncology. Oncol. Lett. 17, 4102–4111 (2019). 30944603 10.3892/ol.2019.10066 PMC6444326

[66] J. M. Campbell, A. Habibalahi, S. Mahbub, M. Gosnell, A. G. Anwer, S. Paton, S. Gronthos, E. Goldys, Non-destructive, label free identification of cell cycle phase in cancer cells by multispectral microscopy of autofluorescence. BMC Cancer 19, 1–11 (2019). 31864316 10.1186/s12885-019-6463-x PMC6925881

[67] M. Wang, H. Z. Kimbrell, A. B. Sholl, D. B. Tulman, K. N. Elfer, T. C. Schlichenmeyer, B. R. Lee, M. Lacey, J. Q. Brown, High-resolution rapid diagnostic imaging of whole prostate biopsies using video-rate fluorescence structured illumination microscopy. Cancer Res. 75, 4032–4041 (2015). 26282168 10.1158/0008-5472.CAN-14-3806 PMC4592466

[68] J. Mertz, Optical sectioning microscopy with planar or structured illumination. Nat. Methods 8, 811–819 (2011). 21959136 10.1038/nmeth.1709

[69] D. R. Yankelevich, D. Ma, J. Liu, Y. Sun, Y. Sun, J. Bec, D. S. Elson, L. Marcu, Design and evaluation of a device for fast multispectral time-resolved fluorescence spectroscopy and imaging. Rev. Sci. Instrum. 85, 034303 (2014). 24689603 10.1063/1.4869037 PMC3971822

[70] J. E. Sorrells, R. R. Iyer, L. Yang, E. M. Martin, G. Wang, H. Tu, M. Marjanovic, S. A. Boppart, Computational photon counting using multithreshold peak detection for fast fluorescence lifetime imaging microscopy. ACS Photonics 9, 2748–2755 (2022). 35996369 10.1021/acsphotonics.2c00505 PMC9389606

[71] L. Shi, C. Zheng, Y. Shen, Z. Chen, E. S. Silveira, L. Zhang, M. Wei, C. Liu, C. de Sena-Tomas, K. Targoff, W. Min, Optical imaging of metabolic dynamics in animals. Nat. Commun. 9, 2995 (2018). 30082908 10.1038/s41467-018-05401-3 PMC6079036

[72] Y. Tan, H. Lin, J.-X. Cheng, Profiling single cancer cell metabolism via high-content SRS imaging with chemical sparsity. Sci. Adv. 9, eadg6061 (2023). 37585522 10.1126/sciadv.adg6061 PMC10431717

[73] M. T. Martell, N. J. Haven, B. D. Cikaluk, B. S. Restall, E. A. McAlister, R. Mittal, B. A. Adam, N. Giannakopoulos, L. Peiris, S. Silverman, J. Deschenes, X. Li, R. J. Zemp, Deep learning-enabled realistic virtual histology with ultraviolet photoacoustic remote sensing microscopy. Nat. Commun. 14, 5967 (2022). 10.1038/s41467-023-41574-2 PMC10519961 37749108

[74] M. Boktor, B. R. Ecclestone, V. Pekar, D. Dinakaran, J. R. Mackey, P. Fieguth, P. Haji Reza, Virtual histological staining of label-free total absorption photoacoustic remote sensing (TA-PARS). Sci. Rep. 12, 10296 (2022). 35717539 10.1038/s41598-022-14042-y PMC9206643

[75] M. Boktor, J. E. Tweel, B. R. Ecclestone, J. A. Ye, P. Fieguth, P. H. Reza, Multi-channel feature extraction for virtual histological staining of photon absorption remote sensing images. arXiv:2307.01824 [eess.IV] (2023). 10.1038/s41598-024-52588-1 PMC10805725 38263394

[76] J. E. Tweel, B. R. Ecclestone, M. Boktor, J. A. T. Simmons, P. Fieguth, P. H. Reza, Virtual histology with photon absorption remote sensing using a cycle-consistent generative adversarial network with weakly registered pairs. arXiv:2306.08583 [physics.med-ph] (2023).

[77] F. Fereidouni, Z. T. Harmany, M. Tian, A. Todd, J. A. Kintner, J. D. McPherson, A. D. Borowsky, J. Bishop, M. Lechpammer, S. G. Demos, R. Levenson, Microscopy with ultraviolet surface excitation for rapid slide-free histology. Nat. Biomed. Eng. 1, 957–966 (2017). 31015706 10.1038/s41551-017-0165-y PMC6223324

[78] A. K. Glaser, N. P. Reder, Y. Chen, E. F. McCarty, C. Yin, L. Wei, Y. Wang, L. D. True, J. T. Liu, Light-sheet microscopy for slide-free non-destructive pathology of large clinical specimens. Nat. Biomed. Eng. 1, 1–10 (2017). 10.1038/s41551-017-0084 PMC5940348 29750130

[79] W. Xie, A. K. Glaser, F. Vakar-Lopez, J. L. Wright, N. P. Reder, J. T. Liu, L. D. True, Diagnosing 12 prostate needle cores within an hour of biopsy via open-top light-sheet microscopy. J. Biomed. Opt. 25, 126502–126502 (2020). 33325186 10.1117/1.JBO.25.12.126502 PMC7744172

[80] Y. K. Tao, D. Shen, Y. Sheikine, O. O. Ahsen, H. H. Wang, D. B. Schmolze, N. B. Johnson, J. S. Brooker, A. E. Cable, J. L. Connolly, J. G. Fijimoto, Assessment of breast pathologies using nonlinear microscopy. Proc. Natl. Acad. Sci. U.S.A. 111, 15304–15309 (2014). 25313045 10.1073/pnas.1416955111 PMC4217415

[81] L. C. Cahill, Y. Wu, T. Yoshitake, C. Ponchiardi, M. G. Giacomelli, A. A. Wagner, S. Rosen, J. G. Fujimoto, Nonlinear microscopy for detection of prostate cancer: Analysis of sensitivity and specificity in radical prostatectomies. Mod. Pathol. 33, 916–923 (2020). 31745288 10.1038/s41379-019-0408-4 PMC7195230

[82] Y. Sun, S. You, X. Du, A. Spaulding, Z. G. Liu, E. J. Chaney, D. R. Spillman Jr., M. Marjanovic, H. Tu, S. A. Boppart, Real-time three-dimensional histology-like imaging by label-free nonlinear optical microscopy. Quant. Imaging Med. Surg. 10, 2177–2190 (2020). 33139997 10.21037/qims-20-381 PMC7547261

[83] D. A. Orringer, B. Pandian, Y. S. Niknafs, T. C. Hollon, J. Boyle, S. Lewis, M. Garrard, S. L. Hervey-Jumper, H. J. Garton, C. O. Maher, J. A. Heth, O. Sagher, D. A. Wilkinson, M. Snuderl, S. Venneti, S. H. Ramkissoon, K. A. Mc Fadden, A. Fisher-Hubbard, A. P. Lieberman, T. D. Johnson, X. S. Xie, J. K. Trautman, C. W. Freudiger, S. Camelo-Piragua, Rapid intraoperative histology of unprocessed surgical specimens via fibre-laser-based stimulated Raman scattering microscopy. Nat. Biomed. Eng. 1, 0027 (2017). 28955599 10.1038/s41551-016-0027 PMC5612414

[84] T. C. Hollon, B. Pandian, A. R. Adapa, E. Urias, A. V. Save, S. S. S. Khalsa, D. G. Eichberg, R. S. D’Amico, Z. U. Farooq, S. Lewis, P. D. Petridis, T. Marie, A. H. Shah, H. J. L. Garton, C. O. Maher, J. A. Heth, E. L. M. Kean, S. E. Sullivan, S. L. Hervey-Jumper, P. G. Patil, B. G. Thompson, O. Sagher, G. M. Mc Khann II, R. J. Komotar, M. E. Ivan, M. Snuderl, M. L. Otten, T. D. Johnson, M. B. Sisti, J. N. Bruce, K. M. Muraszko, J. Trautman, C. W. Freudiger, P. Canoll, H. Lee, S. Camelo-Piragua, D. A. Orringer, Near real-time intraoperative brain tumor diagnosis using stimulated Raman histology and deep neural networks. Nat. Med. 26, 52–58 (2020). 31907460 10.1038/s41591-019-0715-9 PMC6960329

[85] T. T. Wong, R. Zhang, P. Hai, C. Zhang, M. A. Pleitez, R. L. Aft, D. V. Novack, L. V. Wang, Fast label-free multilayered histology-like imaging of human breast cancer by photoacoustic microscopy. Sci. Adv. 3, e1602168 (2017). 28560329 10.1126/sciadv.1602168 PMC5435415

[86] T. Imai, J. Shi, T. T. Wong, L. Li, L. Zhu, L. V. Wang, High-throughput ultraviolet photoacoustic microscopy with multifocal excitation. J. Biomed. Opt. 23, 036007 (2018). 29546734 10.1117/1.JBO.23.3.036007 PMC5852316

[87] R. Cao, S. D. Nelson, S. Davis, Y. Liang, Y. Luo, Y. Zhang, B. Crawford, L. V. Wang, Label-free intraoperative histology of bone tissue via deep-learning-assisted ultraviolet photoacoustic microscopy. Nat. Biomed. Eng. 7, 124–134 (2023). 36123403 10.1038/s41551-022-00940-z PMC10321243

[88] L. Kang, X. Li, Y. Zhang, T. T. Wong, Deep learning enables ultraviolet photoacoustic microscopy based histological imaging with near real-time virtual staining. Photoacoustics 25, 100308 (2022). 34703763 10.1016/j.pacs.2021.100308 PMC8521289

[89] Y. Zhang, L. Kang, I. H. Wong, W. Dai, X. Li, R. C. Chan, M. K. Hsin, T. T. Wong, High-throughput, label-free and slide-free histological imaging by computational microscopy and unsupervised learning. Adv. Sci. 9, 2102358 (2022). 10.1002/advs.202102358 PMC8805566 34747142

[90] S. Soltani, A. Ojaghi, H. Qiao, N. Kaza, X. Li, Q. Dai, A. O. Osunkoya, F. E. Robles, Prostate cancer histopathology using label-free multispectral deep-UV microscopy quantifies phenotypes of tumor aggressiveness and enables multiple diagnostic virtual stains. Sci. Rep. 12, 9329 (2022). 35665770 10.1038/s41598-022-13332-9 PMC9167293

[91] M. Ragazzi, S. Piana, C. Longo, F. Castagnetti, M. Foroni, G. Ferrari, G. Gardini, G. Pellacani, Fluorescence confocal microscopy for pathologists. Mod. Pathol. 27, 460–471 (2014). 24030744 10.1038/modpathol.2013.158

[92] J. Pérez-Anker, S. Ribero, O. Yélamos, A. Garcia-Herrera, L. Alos, B. Alejo, M. Combalia, D. Moreno-Ramirez, J. Malvehy, S. Puig, Basal cell carcinoma characterization using fusion ex vivo confocal microscopy: A promising change in conventional skin histopathology. Br. J. Dermatol. 182, 468–476 (2020). 31220341 10.1111/bjd.18239 PMC6923630

[93] J. Li, J. Garfinkel, X. Zhang, D. Wu, Y. Zhang, K. De Haan, H. Wang, T. Liu, B. Bai, Y. Rivenson, G. Rubinstein, P. O. Scumpia, A. Ozcan, Biopsy-free in vivo virtual histology of skin using deep learning. Light Sci. Appl. 10, 233 (2021). 34795202 10.1038/s41377-021-00674-8 PMC8602311

[94] B. E. Bejnordi, N. Timofeeva, I. Otte-Höller, N. Karssemeijer, J. A. van der Laak, Quantitative analysis of stain variability in histology slides and an algorithm for standardization, in *Medical Imaging 2014: Digital Pathology* (International Society for Optics and Photonics, 2014), vol. 9041, pp. 904108.

[95] K. B. Patel, W. Liang, M. J. Casper, V. Voleti, W. Li, A. J. Yagielski, H. T. Zhao, C. Perez Campos, G. S. Lee, J. M. Liu, E. Philipone, A. J. Yoon, K. P. Olive, S. M. Coley, E. M. C. Hillman, High-speed light-sheet microscopy for the in-situ acquisition of volumetric histological images of living tissue. Nat. Biomed. Eng. 6, 569–583 (2022). 35347275 10.1038/s41551-022-00849-7 PMC10353946

[96] T. Yoshitake, M. G. Giacomelli, L. M. Quintana, H. Vardeh, L. C. Cahill, B. E. Faulkner-Jones, J. L. Connolly, D. Do, J. G. Fujimoto, Rapid histopathological imaging of skin and breast cancer surgical specimens using immersion microscopy with ultraviolet surface excitation. Sci. Rep. 8, 1–12 (2018). 29540700 10.1038/s41598-018-22264-2 PMC5852098

[97] W. Xie, Y. Chen, Y. Wang, L. Wei, C. Yin, A. K. Glaser, M. E. Fauver, E. J. Seibel, S. M. Dintzis, J. C. Vaughan, N. P. Reder, J. T. C. Liu, Microscopy with ultraviolet surface excitation for wide-area pathology of breast surgical margins. J. Biomed. Opt. 24, 026501 (2019). 30737911 10.1117/1.JBO.24.2.026501 PMC6368047

[98] E. Olson, M. J. Levene, R. Torres, Multiphoton microscopy with clearing for three dimensional histology of kidney biopsies. Biomed. Opt. Express 7, 3089–3096 (2016). 27570700 10.1364/BOE.7.003089 PMC4986816

[99] Y. Rivenson, H. Wang, Z. Wei, K. de Haan, Y. Zhang, Y. Wu, H. Günaydın, J. E. Zuckerman, T. Chong, A. E. Sisk, L. M. Westbrook, W. D. Wallace, A. Ozcan, Virtual histological staining of unlabelled tissue-autofluorescence images via deep learning. Nat. Biomed. Eng. 3, 466–477 (2019). 31142829 10.1038/s41551-019-0362-y

[100] Y. Winetraub, E. Yuan, I. Terem, C. Yu, W. Chan, H. Do, S. Shevidi, M. Mao, J. Yu, M. Hong, E. Blankenberg, K. E. Rieger, S. Chu, S. Aasi, K. Y. Sarin, A. de la Zerda, OCT2Hist: Non-invasive virtual biopsy using optical coherence tomography. medRxiv 2021.03.31.21254733 [Preprint] (2021). 10.1101/2021.03.31.21254733.

[101] D.-K. Yao, K. Maslov, K. K. Shung, Q. Zhou, L. V. Wang, In vivo label-free photoacoustic microscopy of cell nuclei by excitation of DNA and RNA. Opt. Lett. 35, 4139–4141 (2010). 21165116 10.1364/OL.35.004139 PMC3048585

[102] T. T. Wong, R. Zhang, C. Zhang, H.-C. Hsu, K. I. Maslov, L. Wang, J. Shi, R. Chen, K. K. Shung, Q. Zhou, L. V. Wang, Label-free automated three-dimensional imaging of whole organs by microtomy-assisted photoacoustic microscopy. Nat. Commun. 8, 1386 (2017). 29123109 10.1038/s41467-017-01649-3 PMC5680318

[103] H. Kim, J. W. Baik, S. Jeon, J. Y. Kim, C. Kim, PAExM: Label-free hyper-resolution photoacoustic expansion microscopy. Opt. Lett. 45, 6755–6758 (2020). 33325889 10.1364/OL.404041

[104] X. Li, L. Kang, Y. Zhang, T. T. W. Wong, High-speed label-free ultraviolet photoacoustic microscopy for histology-like imaging of unprocessed biological tissues. Opt. Lett. 45, 5401–5404 (2020). 33001904 10.1364/OL.401643

[105] C. Zhang, Y. S. Zhang, D.-K. Yao, Y. Xia, L. V. Wang, Label-free photoacoustic microscopy of cytochromes. J. Biomed. Opt. 18, 020504 (2013). 23370407 10.1117/1.JBO.18.2.020504 PMC3560837

[106] V. Gorti, F. E. Robles, Characterizing UV-induced photo-damage to improve long-term, dynamic live cell imaging with UV microscopy, in *Label-free Biomedical Imaging and Sensing (LBIS) 2024* (SPIE, 2024), vol. 12854, pp. 43–46.

[107] L. Shi, L. Lu, G. Harvey, T. Harvey, A. Rodriguez-Contreras, R. R. Alfano, Label-free fluorescence spectroscopy for detecting key biomolecules in brain tissue from a mouse model of Alzheimer’s disease. Sci. Rep. 7, 2599 (2017). 28572632 10.1038/s41598-017-02673-5 PMC5454017

