# Supplementary material for: Metabolic light absorption, scattering, and emission (MetaLASE) microscopy
Source: Sci Adv. 2024 Oct 18;10(42):eadl5729. doi: 10.1126/sciadv.adl5729 (PMC11488571; doi:10.1126/sciadv.adl5729)
Supplement: Supplementary file 1 — Supplementary Text Figs. S1 to S9 References [file sciadv.adl5729_sm.pdf]

Science Advances
AAAS

# Supplementary Materials for

## Metabolic light absorption, scattering, and emission (MetaLASE) microscopy

Brendon S. Restall *et al.*

Corresponding author: Roger J. Zemp, rzemp@ualberta.ca

*Sci. Adv.* **10**, eadl5729 (2024)
DOI: 10.1126/sciadv.adl5729

**This PDF file includes:**

Supplementary Text
Figs. S1 to S9
References

# 1 Virtual Histology Modalities

## 1.1 Fluorescent Stain-Based Methods

Many virtual histology modalities are capable of slide-free H&E-like imaging in thick tissue samples, but still rely on fluorescent s taining w ith a gents s uch a s a cridine o range, proflavine, DAPI, propidium iodide, Hoechst, and rhodamine for contrast to cell nuclei and other histological features. The need for exogenous labeling and in some cases optical clearing agents can add minutes to the tissue processing workflow, introduce staining variability (*94*), and potentially interfere with downstream analysis including immunohistochemistry, molecular assays, or spe-cial staining of the same tissue section. Toxicity of exogenous agents and regulatory barriers may also present challenges for *in vivo* applications compared to label-free approaches.

Wide field of view imaging architectures reported for fluorescence-based virtual histology include light-sheet microscopy (*78, 95*) and microscopy with ultraviolet surface excitation (MUSE) (*77*). These approaches confer the advantage of exceptional imaging speeds due to par-allelized, camera-based detection. Light-sheet microscopy additionally offers volumetric imag-ing capabilities, though this typically requires optical clearing, and data management require-ments may challenge clinical translation. Alternatively, MUSE offers a simple and economical widefield implementation for slide-free surface imaging of thick specimens. However, the re-liance on UV penetration (which is tissue-dependent) rather than optical focusing for depth discrimination can lead to confusion of cell layers and consequently unclear interpretations in margin analysis (*96, 97*). In general, these widefield methods typically trade-off some lateral and axial resolution compared to laser scanning approaches including confocal fluorescence microscopy (*91*), nonlinear microscopy (*80, 98*), or some reported label-free imaging technologies.

## 1.2 Label-Free Methods

Label-free virtual histology methods avoiding the need for staining can simplify workflows for intraoperative margin assessment and surgical guidance, offering advantages for many applications. Such methods rely on various intrinsic tissue contrasts including autofluorescence, multiharmonic generation, elastic or inelastic scattering, or non-radiative absorption-induced thermoelastic expansion.

Reported autofluorescence-based modalities include a variety of implementations such as conventional transillumination microscopy (*99*), structured illumination approaches like computational high-throughput autofluroescence microscopy by pattern illumination (CHAMP) (*89*), confocal laser scanning or multiphoton excitation autofluorescence microscopes, and widefield approaches such as swept confocally-aligned planar excitation light-sheet microscopy (MediS-CAPE) (*95*). Simultaneous label-free autofluorescence-multiharmonic (SLAM) microscopy adds multiharmonic generation contrast using a single excitation light source, revealing rich struc-tural and functional information in the tumor microenvironment (*82*). However, as with other autofluorescence methods, achieving positive nuclei contrast similar to a hematoxylin stain is an outstanding challenge.

Optical imaging methods based on elastic scattering have been assisted by deep learning to produce virtual histology, including reflectance confocal microscopy (RCM) (*93*), and optical coherence tomography (*100*), which generates fast cross-sectional or 3D volumetric scattering images, but lacks molecular specificity and the resolution required to reveal sub-cellular detail. Stimulated Raman scattering (SRS) is a nonlinear inelastic scattering process, where Raman shifts related to $CH_2$ and $CH_3$ vibrational modes and their combinations have been used to generate virtual histology (*83, 84*). However, scan speeds are currently limited, and the correspondence of these contrasts to gold standard hematoxylin and eosin staining is not ideal for maximal realism. Multispectral deep-UV microscopy has also been used for virtual histolog-

ical imaging with promising results, though this system operates in transmission-mode and is therefore limited to thin samples (*90*).

Similar to UV-PARS virtual histology, ultraviolet photoacoustic microscopy (UV-PAM) relies on non-radiative, absorption-induced thermoelastic expansion to provide label-free virtual histology (*85, 86, 88, 101–104*). In both cases, DNA in cell nuclei is the chromophore providing positive cell nuclei contrast. However, rather than using an all-optical, non-contact system architecture that detects absorption-induced reflectance modulations in a co-focused interrogation beam signal, conventional UV-PAM utilizes transducer-based detection of propagating ultrasonic waves, and therefore requires immersion or acoustic coupling to the sample. This can lead to reduced scan speeds, and sub-optimal lateral resolution when imaging thick tissues in reflection-mode, where coupling setups can introduce optical aberrations and restrict focusing numerical apertures. Whereas UV-PARS virtual histology can produce surface imagery in thick tissues by flattening the specimen against a UV transparent coverslip, this is not always possible for reflection-mode UV-PAM where acoustic transparency is a factor, and slow contour scanning must be performed to accommodate irregular sample surfaces. To produce a complete virtual H&E stain, cytoplas-mic contrast can been added via additional cytochrome-targeted excitation wavelengths (*105*), or by deep learning enabled inference from weaker cytoplasmic absorption at the 266 nm DNA-targeted excitation wavelength (*88*). However, limitations include weaker signal-to-noise ratios in these cytoplasmic contrast mechanisms, or resolution mismatches in the hematoxylin-like and eosin-like data channels, which may compromise the accuracy or histological realism of results.

## 1.3 Cell viability study

When using UV-C light, phototoxicity resulting in cell damage or death is a concern. In a previous study, cell death occurred with 4.8 mJ of energy deposited on average (*106*). Alternatively,

as we scan line-by-line over the imaging plane, the energy deposited in the cells is over a substantial time frame of 5 s, leading to an average irradiance of 11.2 $\mu$W/$\mu$m$^2$. However, when the cell is under direct light exposure it has an irradiance of 1.12 mW/$\mu$m$^2$. During our scans based on average cell area, stage step size, our 400kHz pulse repetition rate, and our 350 pJ pulse energy, we deposit around 5.6 $\mu$J on each cell or 3 orders of magnitude under the lowest energy for cell death.

To quantify cell viability we observed the effect of UV exposure on cells at different pulse energies, and without UV irradiation for control. These results are shown in Supplementary Information Fig. S7. We see a modest decrease of 5.6 % in cell viability on our low power metabolic imaging set point, but when using a higher laser power we see a large decrease of 64.4 % in cell count. Cell death was also observed in the control likely due to cell stresses such as removing the media plate from incubation during imaging sessions.

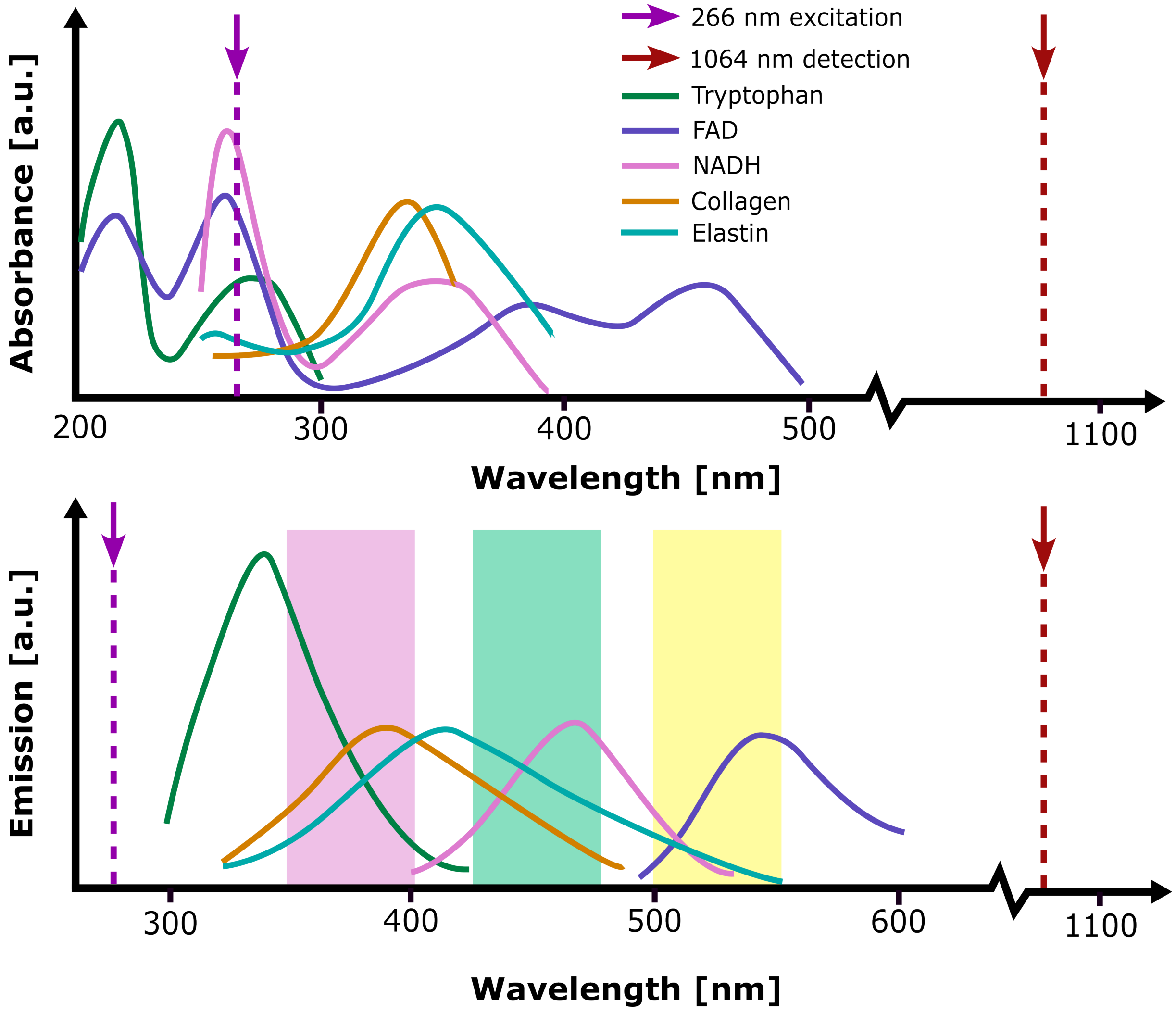


**Fig. S 1. Autofluorescence absorption and emission bands with system excitation and collection bands.** Excitation and emission spectra for several autofluorescent biomolecules of interest including the redox coenzymes FAD and NAD(P)H, and structural proteins collagen and elastin. The MetaLASE system excitation and interrogation wavelengths are denoted with a dashed line. The MetaLASE system fluorescent emission filter bands are indicated in pink (collagen), green (NAD(P)H), and yellow (FAD). Data taken from Shi *et al.* (*107*).

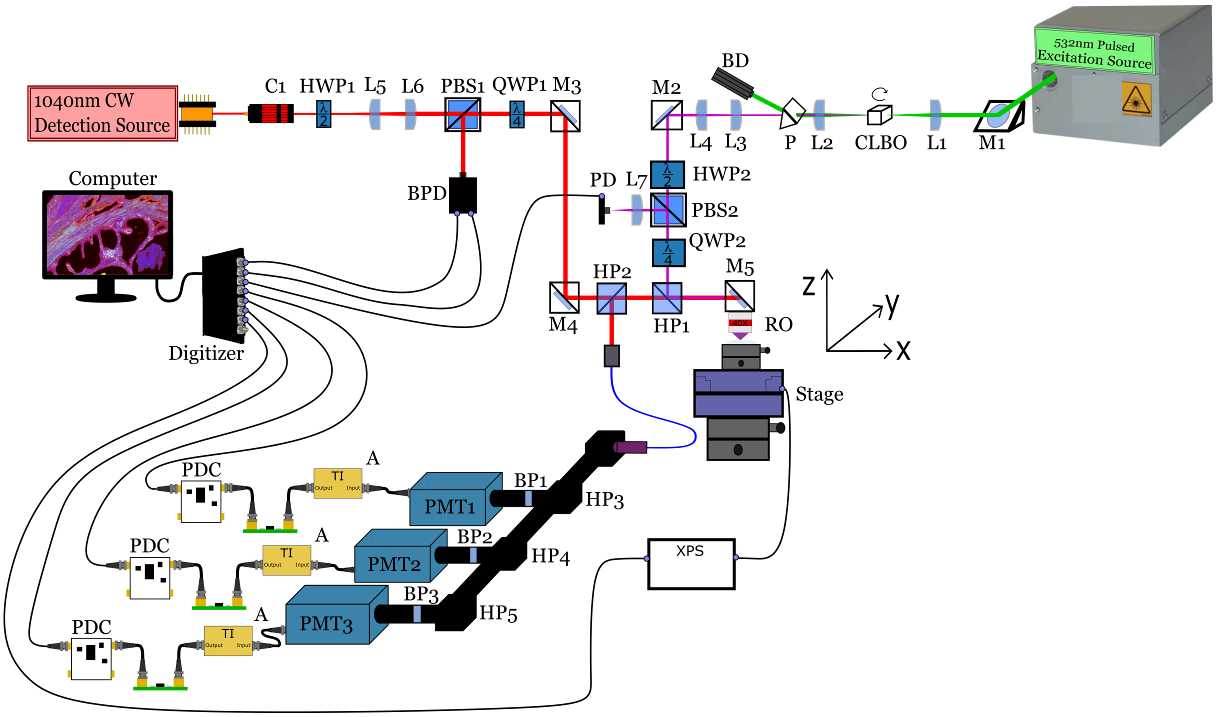


**Fig. S 2. MetaLASE full system diagram.** A, amplifier; C, collimator; HWP, half-wave plate; L, lens; PBS, polarizing beam splitter; QWP, quarter-wave plate; M, mirror; BPD, balanced photodiode; PD, photodiode; PDC, peak detection circuit; HP, high-pass dichroic filter; BD, beam dump; P, prism; CLBO, caesium lithium borate; RO, reflective objective; BP, bandpass filter; PMT, photomultiplier tube; TI, transimpedance amplifier.

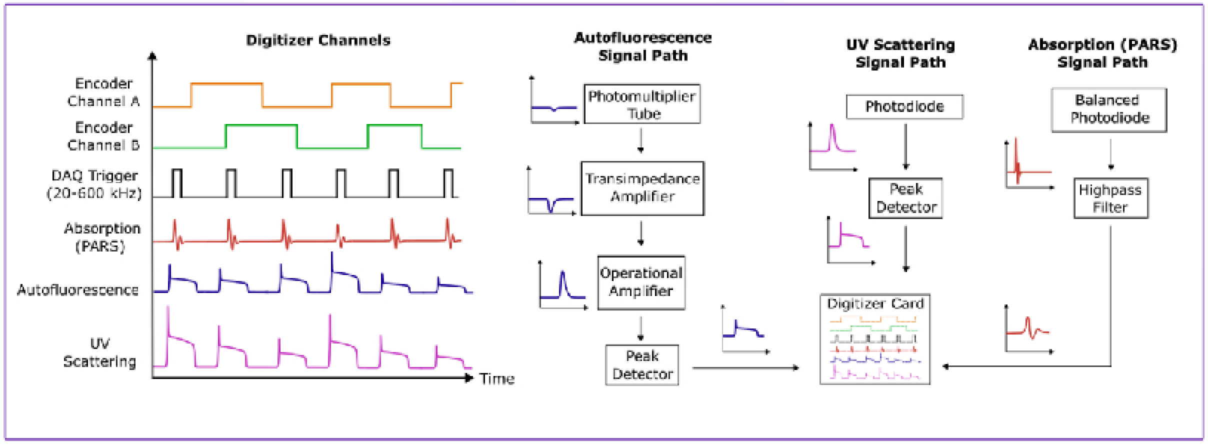


**Fig. S 3. MetaLASE signal processing and digitizer timing diagrams.** Left: timing diagram of representative digitizer channels captured during MetaLASE imaging. Right: diagrams showing how captured absorption, scattering, and autofluorescence emission signals are processed for adequate digitizer card sampling. In the autofluorescence signal path, the photomultiplier tube signal is amplified and then inverted before being sample and held with a peak detector; in the UV scattering signal path, the photodiode signal is sample and held with a peak detector; and in the absorption (PARS) signal path, the balanced photodiode modulated interrogation signal is highpass filtered.

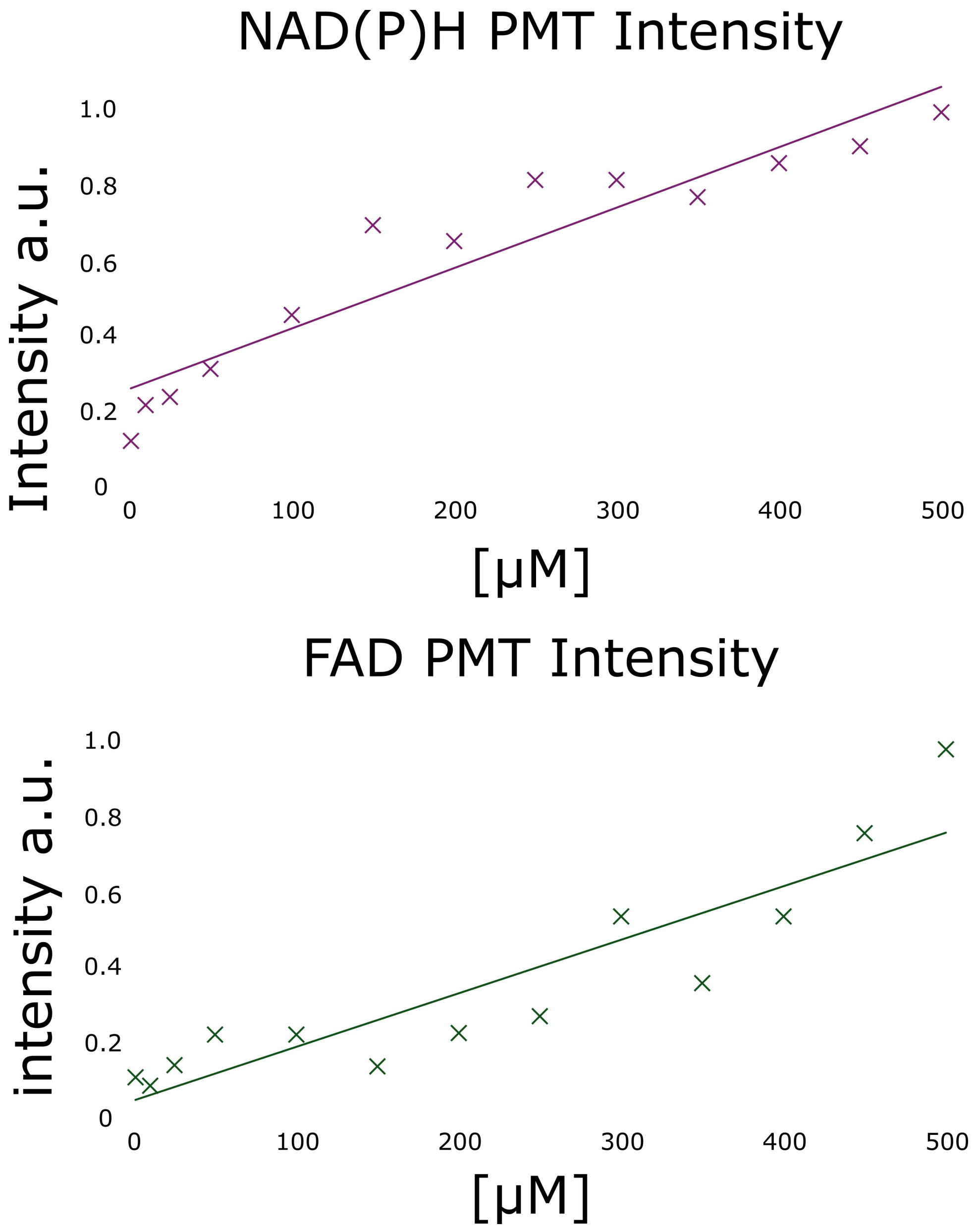


**Fig. S 4. NAD(P)H & FAD standard dilution calibration curves.** Photomultiplier tube voltage measurements for different NAD(P)H and FAD dilution concentrations ranging from 1 $\mu$M - 500 $\mu$M. An intensity vs concentration scatter plot is shown (purple and green crosses for NAD(P)H and FAD respectively) and a linear line is fit with corresponding $R^2$ values of 0.891 and 0.821 for NAD(P)H and FAD respectively.

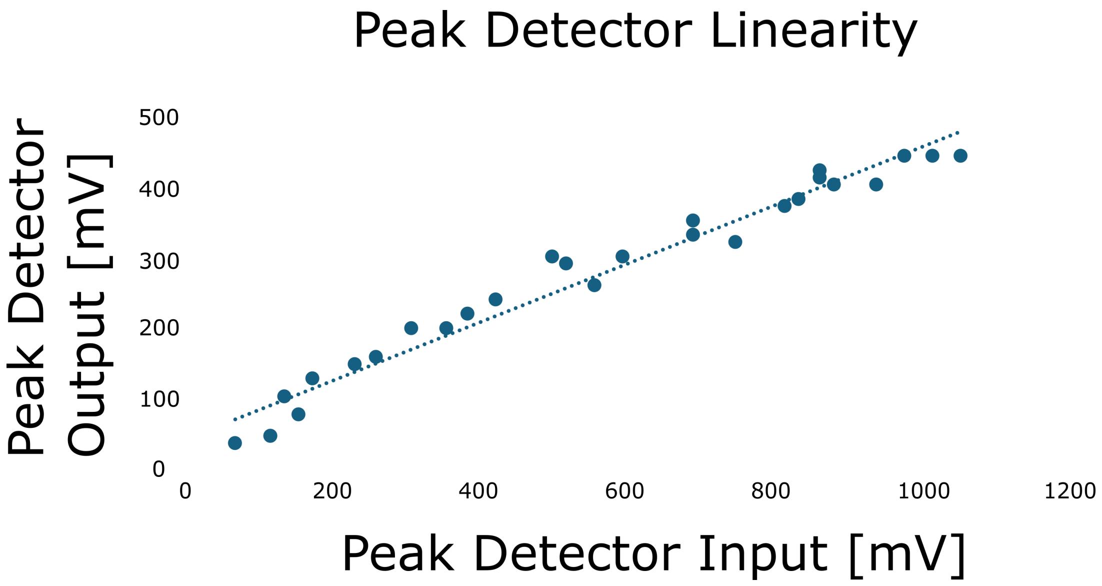


**Fig. S 5. Peak detector linearity for input and output voltages.** Measured peak detector output voltage for different input PMT voltages. A linear fit is added to the scatter plot with a calculated $R^2$ value of 0.965.

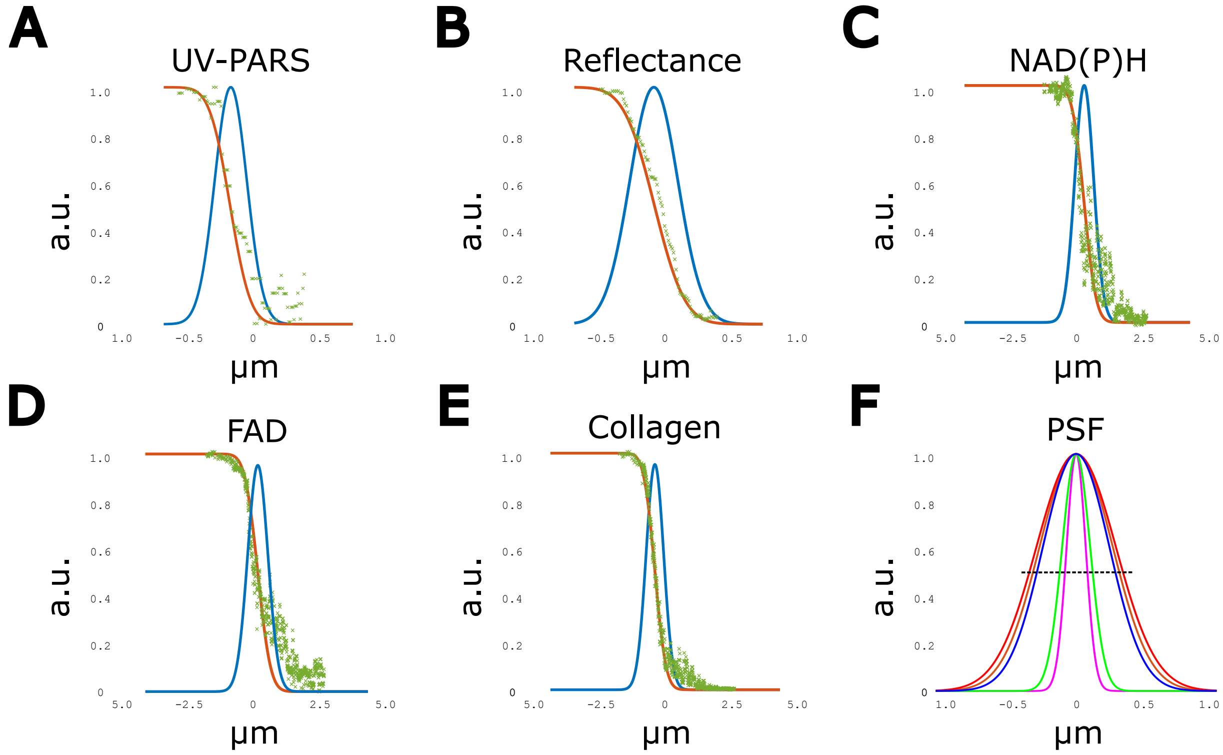


**Fig. S 6. MetaLASE resolution calculations and comparison.** Scatter plots showing intensity vs position (green crosses) for each MetaLASE channel where an edge of an imaging phantom was imaged. A fitted edge spread function (orange) and the derivative of the edge spread function corresponding to the point spread function (blue) is overlaid. The full width at half maxima of these point spread functions is used to determine lateral resolutions of: **A**) 0.945 $\mu$m for collagen autofluorescence, **B**) 1.045 $\mu$m for NAD(P)H autofluorescence, **C**) 1.134 $\mu$m for FAD autofluorescence, **D**) 0.400 $\mu$m for UV reflectance, and **E**) 0.273 $\mu$m for UV-PARS. **F**) Shows an overlay of the point spread functions for comparison: magenta for UV-PARS, green for UV reflectance, blue for collagen autofluorescence, orange for NAD(P)H autofluorescence, and red for FAD autofluorescence.

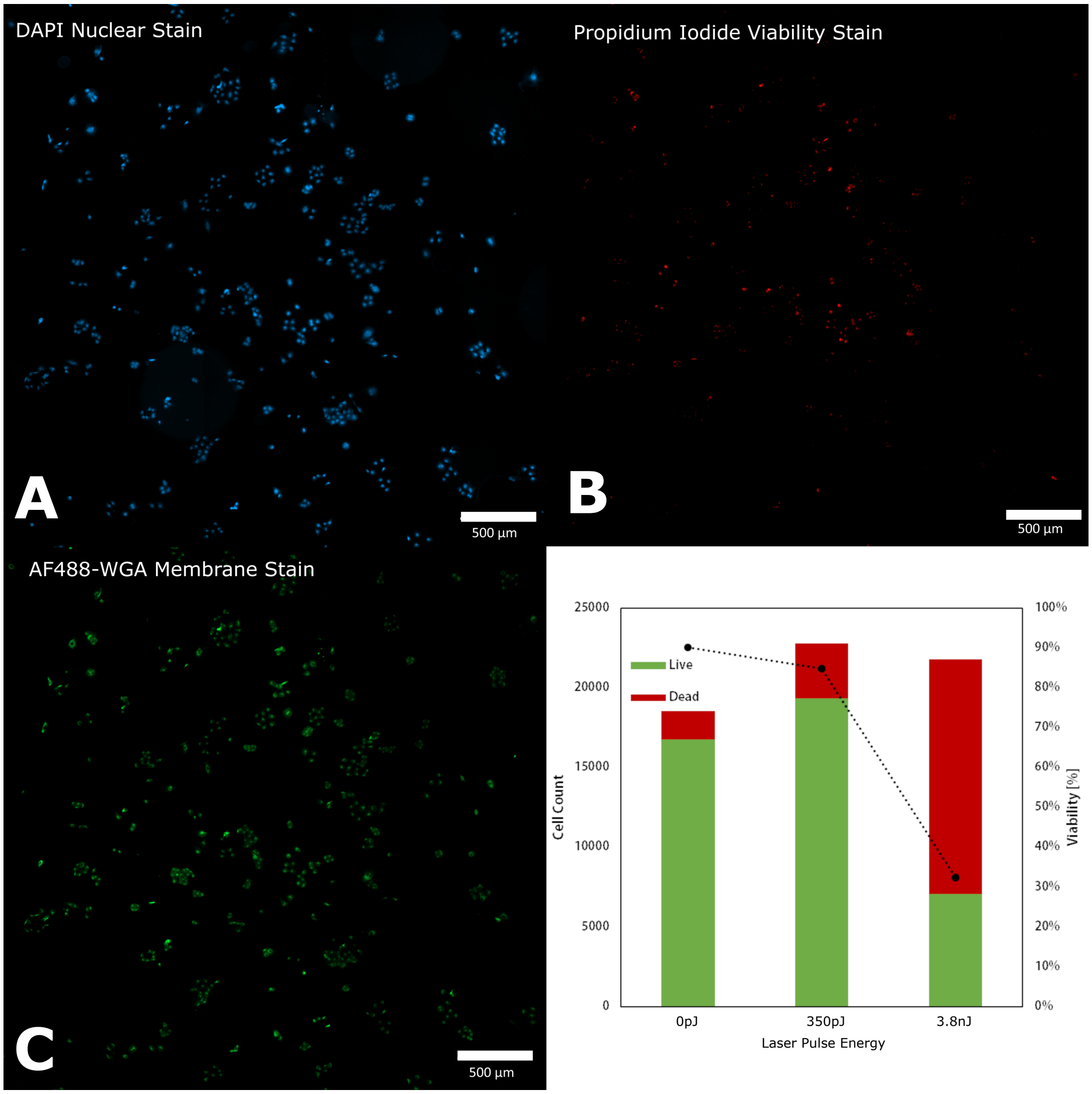


**Fig. S 7. Cell viability with three different irradiance exposures.** Cells were stained to differentiate the live and dead cells from our three different irradiance exposure conditions, which consisted of no exposure (control), 350 pJ pulse energies (140 $\mu$mW average power), and 3.8 nJ pulse energies (1.53 mW average power). The entirety of samples were imaged with these UV excitation laser parameters as normal over the course of 10 minutes. Fluorescence images are shown for: **A**) DAPI nuclear stain, **B**) propidium iodide viability stain, and **C**) AF488-WGA membrane stain. We observed a 5.6% decrease in cell viability on our low power metabolic imaging set point and a decrease of 64.4% in cell viability for our higher laser set point.

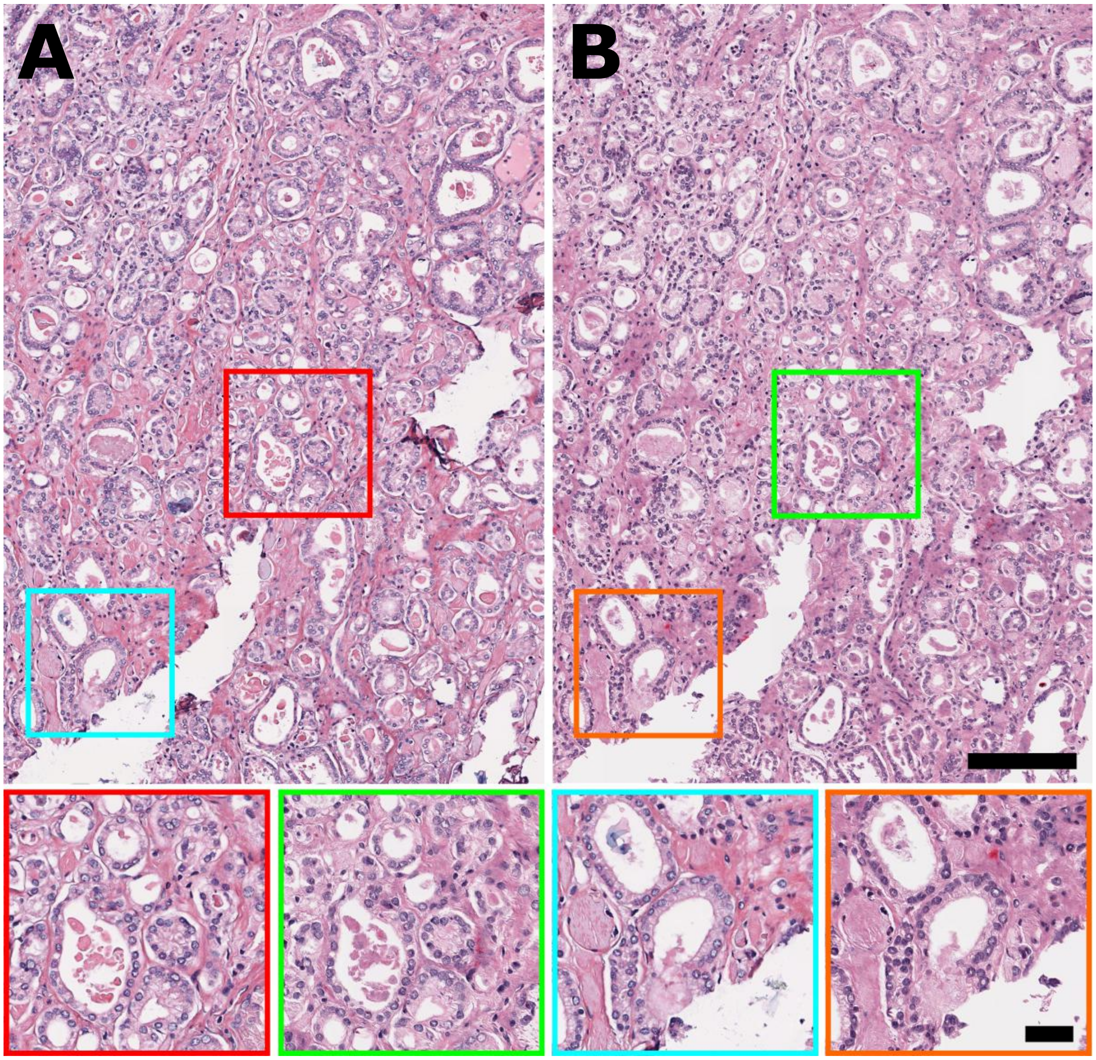


**Fig. S 8. Virtual and true H&E staining comparisons.** **A**) True H&E-stained brightfield histology image of a sectioned radical prostatectomy specimen. **B**) Corresponding virtual histology image taken with our MetaLASE system. Scale bar: 200 $\mu$m. Colored square inset scale bars: 50 $\mu$m.

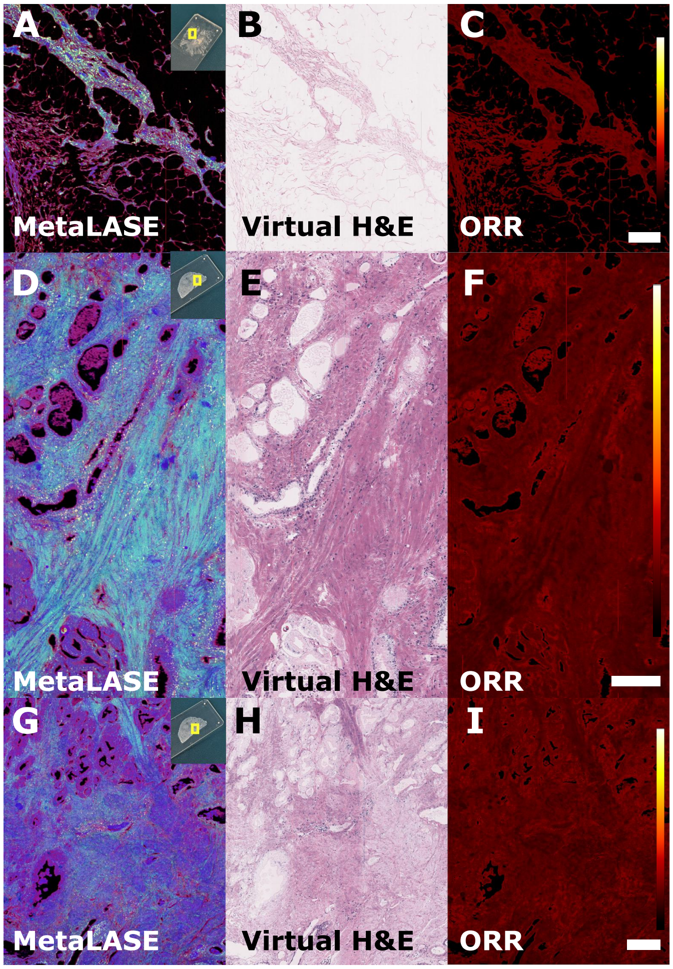


**Fig. S 9. Additional thin FFPE breast and prostate tissues showing MetaLASE, virtual histology, and ORR images.** MetaLASE images of FFPE lumpectomy (**A**) and radical prostatectomy specimens (**D** and **G**MetaLASE resolution calculations and comparison). MetaLASE images are shown in **A**, **D**, and **G**, virtual histology images are shown in **B**, **E**, and **H**, and ORR maps are shown in **C**, **F**, and **I**. ORR color bars correspond to a linear scale between 0 and 1. Scale bars: 200 $\mu$m.

## REFERENCES AND NOTES


1. V. Di Gialleonardo, D. M. Wilson, K. R. Keshari, The potential of metabolic imaging. *Semin. Nucl. Med.* **46**, 28–39 (2016).

2. O. I. Kolenc, K. P. Quinn, Evaluating cell metabolism through autofluorescence imaging of NAD(P)H and FAD. *Antioxid. Redox Signal.* **30**, 875–889 (2019).

3. I. Georgakoudi, K. P. Quinn, Label-free optical metabolic imaging in cells and tissues. *Annu. Rev. Biomed. Eng.* **25**, 413–443 (2023).

4. B. Chance, B. Schoener, R. Oshino, F. Itshak, Y. Nakase, Oxidation-reduction ratio studies of mitochondria in freeze-trapped samples. NADH and flavoprotein fluorescence signals. *J. Biol. Chem.* **254**, 4764–4771 (1979).

5. A. Shiino, M. Haida, B. Beauvoit, B. Chance, Three-dimensional redox image of the normal gerbil brain. *Neuroscience* **91**, 1581–1585 (1999).

6. L. E. Navas, A. Carnero, $NAD^+$ metabolism, stemness, the immune response, and cancer. *Signal Transduct. Target. Ther.* **6**, 2 (2021).

7. M. A. Yaseen, S. Sakadžić, W. Wu, W. Becker, K. A. Kasischke, D. A. Boas, In vivo imaging of cerebral energy metabolism with two-photon fluorescence lifetime microscopy of NADH. *Biomed. Opt. Express* **4**, 307–321 (2013).

8. S. Palmer, K. Litvinova, E. U. Rafailov, G. Nabi, Detection of urinary bladder cancer cells using redox ratio and double excitation wavelengths autofluorescence. *Biomed. Opt. Express* **6**, 977–986 (2015).

9. H. N. Xu, S. Nioka, J. D. Glickson, B. Chance, L. Z. Li, Quantitative mitochondrial redox imaging of breast cancer metastatic potential. *J. Biomed. Opt.* **15**, 036010 (2010).

10. H. N. Xu, J. Tchou, L. Z. Li, Redox imaging of human breast cancer core biopsies: A preliminary investigation. *Acad. Radiol.* **20**, 764–768 (2013).

11. J. Xu, X. Luo, G. Wang, H. Gilmore, A. Madabhushi, A deep convolutional neural network for segmenting and classifying epithelial and stromal regions in histopathological images. *Neurocomputing* **191**, 214–223 (2016).

12. I. Sabdyusheva Litschauer, K. Becker, S. Saghafi, S. Ballke, C. Bollwein, M. Foroughipour, J. Gaugeler, M. Foroughipour, V. Schavelová, V. László, B. Döme, C. Brostjan, W. Weichert, H. U. Dodt, 3D histopathology of human tumours by fast clearing and ultramicroscopy. *Sci. Rep.* **10**, 17619 (2020).

13. L. Waaijer, M. D. Filipe, J. Simons, C. C. van der Pol, T. de Boorder, P. J. van Diest, A. J. Witkamp, Detection of breast cancer precursor lesions by autofluorescence ductoscopy. *Breast Cancer* **28**, 119–129 (2021).

14. M. C. Skala, K. M. Riching, A. Gendron-Fitzpatrick, J. Eickhoff, K. W. Eliceiri, J. G. White, N. Ramanujam, In vivo multiphoton microscopy of NADH and FAD redox states, fluorescence lifetimes, and cellular morphology in precancerous epithelia. *Proc. Natl. Acad. Sci. U.S.A.* **104**, 19494–19499 (2007).

15. B. Lin, S. Urayama, R. M. G. Saroufeem, D. L. Matthews, S. G. Demos, Characterizing the origin of autofluorescence in human esophageal epithelium under ultraviolet excitation. *Opt. Express* **18**, 21074–21082 (2010).

16. R. Cao, H. K. Wallrabe, A. Periasamy, Multiphoton FLIM imaging of NAD(P)H and FAD with one excitation wavelength. *J. Biomed. Opt.* **25**, 014510 (2020).

17. G. Fürtjes, D. Reinecke, N. von Spreckelsen, A.-K. Meißner, D. Rueß, M. Timmer, C. Freudiger, A. Ion-Margineanu, F. Khalid, K. Watrinet, C. Mawrin, A. Chmyrov, R. Goldbrunner, O. Bruns, V. Neuschmelting, Intraoperative microscopic autofluorescence detection and characterization in brain tumors using stimulated Raman histology and two-photon fluorescence. *Front. Oncol.* **13**, 1146031 (2023).

18. N. J. M. Haven, K. L. Bell, P. Kedarisetti, J. D. Lewis, R. J. Zemp, Ultraviolet photoacoustic remote sensing microscopy. *Opt. Lett.* **44**, 3586–3589 (2019).

19. N. J. Haven, P. Kedarisetti, B. S. Restall, R. J. Zemp, Reflective objective-based ultraviolet photoacoustic remote sensing virtual histopathology. *Opt. Lett.* **45**, 535–538 (2020).

20. P. Hajireza, W. Shi, K. Bell, R. J. Paproski, R. J. Zemp, Non-interferometric photoacoustic remote sensing microscopy. *Light Sci. Appl.* **6**, e16278–e16278 (2017).

21. P. H. Reza, K. Bell, W. Shi, J. Shapiro, R. J. Zemp, Deep non-contact photoacoustic initial pressure imaging. *Optica* **5**, 814–820 (2018).

22. N. J. Haven, M. T. Martell, H. Li, J. D. Hogan, R. J. Zemp, Investigating mechanisms of laser pulse-induced reflectivity modulations in photoacoustic remote sensing with a 10 million frames-per-second camera. *Sci. Rep.* **13**, 3751 (2023).

23. B. S. Restall, B. D. Cikaluk, M. T. Martell, N. J. M. Haven, R. Mittal, S. Silverman, L. Peiris, J. Deschenes, B. A. Adam, A. Kinnaird, R. J. Zemp, Fast hybrid optomechanical scanning photoacoustic remote sensing microscopy for virtual histology. *Biomed. Opt. Express* **13**, 39–47 (2022).

24. S. Abbasi, M. Le, B. Sonier, D. Dinakaran, G. Bigras, K. Bell, J. R. Mackey, P. Haji Reza, All-optical reflection-mode microscopic histology of unstained human tissues. *Sci. Rep.* **9**, 13392 (2019).

25. B. Ecclestone, D. Dinakaran, P. H. Reza, Single acquisition label-free histology-like imaging with dual-contrast photoacoustic remote sensing microscopy. *J. Biomed. Opt.* **26**, 056007 (2021).

26. B. S. Restall, N. J. Haven, P. Kedarisetti, M. T. Martell, B. D. Cikaluk, S. Silverman, L. Peiris, J. Deschenes, R. J. Zemp, Virtual hematoxylin and eosin histopathology using simultaneous photoacoustic remote sensing and scattering microscopy. *Opt. Express* **29**, 13864–13875 (2021).

27. P. Kedarisetti, B. S. Restall, N. J. Haven, M. T. Martell, B. D. Cikaluk, J. Deschenes, R. J. Zemp, F-mode ultraviolet photoacoustic remote sensing for label-free virtual H&E histopathology using a single excitation wavelength. *Opt. Lett.* **46**, 3500–3503 (2021).

28. K. Bell, S. Abbasi, D. Dinakaran, M. Taher, G. Bigras, F. K. van Landeghem, J. R. Mackey, P. Haji Reza, Reflection-mode virtual histology using photoacoustic remote sensing microscopy. *Sci. Rep.* **10**, 19121 (2020).

29. N. Pellegrino, B. R. Ecclestone, D. Dinakaran, F. van Landeghem, P. Fieguth, P. H. Reza, Time-domain feature extraction for target specificity in photoacoustic remote sensing microscopy. *Opt. Lett.* **47**, 3952–3955 (2022).

30. M. T. Martell, N. J. Haven, R. J. Zemp, Multimodal imaging with spectral-domain optical coherence tomography and photoacoustic remote sensing microscopy. *Opt. Lett.* **45**, 4859–4862 (2020).

31. B. R. Ecclestone, Z. Hosseinaee, N. Abbasi, K. Bell, D. Dinakaran, J. R. Mackey, P. Haji Reza, Three-dimensional virtual histology in unprocessed resected tissues with photoacoustic remote sensing (PARS) microscopy and optical coherence tomography (OCT). *Sci. Rep.* **11**, 13723 (2021).

32. M. T. Martell, N. J. Haven, R. J. Zemp, Fiber-based photoacoustic remote sensing microscopy and spectral-domain optical coherence tomography with a dual-function 1050-nm interrogation source. *J. Biomed. Opt.* **26**, 066502 (2021).

33. B. S. Restall, P. Kedarisetti, N. J. Haven, M. T. Martell, R. J. Zemp, Multimodal 3D photoacoustic remote sensing and confocal fluorescence microscopy imaging. *J. Biomed. Opt.* **26**, 096501 (2021).

34. N. J. Haven, M. T. Martell, B. D. Cikaluk, B. S. Restall, E. McAlister, S. Silverman, L. Peiris, J. Deschenes, X. Li, R. J. Zemp, Virtual histopathology with ultraviolet scattering and photoacoustic remote sensing microscopy. *Opt. Lett.* **46**, 5153–5156 (2021).

35. M. T. Martell, N. J. Haven, B. D. Cikaluk, B. S. Restall, E. A. McAlister, R. Mittal, B. A. Adam, N. Giannakopoulos, L. Peiris, S. Silverman, J. Deschenes, X. Li, R. J. Zemp, Deep learning-enabled realistic virtual histology with ultraviolet photoacoustic remote sensing microscopy. *Nat. Commun.* **14**, 5967 (2023).

36. M. A. Yaseen, J. Sutin, W. Wu, B. Fu, H. Uhlirova, A. Devor, D. A. Boas, S. Sakadžić, Fluorescence lifetime microscopy of nadh distinguishes alterations in cerebral metabolism in vivo. *Biomed. Opt. Express* **8**, 2368–2385 (2017).

37. C. T. Hensley, A. T. Wasti, R. J. DeBerardinis, Glutamine and cancer: Cell biology, physiology, and clinical opportunities. *J. Clin. Invest.* **123**, 3678–3684 (2013).

38. A. Sánchez-Hernández, C. M. Polleys, I. Georgakoudi, Formalin fixation and paraffin embedding interfere with preservation of optical metabolic assessments based on endogenous NAD(P)H and FAD two photon excited fluorescence. bioRxiv 2023.06.16.545363 [Preprint] (2023). https://doi.org/10.1101/2023.06.16.545363.

39. L. Z. Li, M. Masek, T. Wang, H. N. Xu, S. Nioka, J. A. Baur, T. M. Ragan, Two-photon autofluorescence imaging of fixed tissues: Feasibility and potential values for biomedical applications. *Adv. Exp. Med. Biol.* **1232**, 375–381 (2020).

40. H. N. Xu, H. Zhao, K. Chellappa, J. G. Davis, S. Nioka, J. A. Baur, L. Z. Li, Optical redox imaging of fixed unstained muscle slides reveals useful biological information. *Mol. Imaging Biol.* **21**, 417–425 (2019).

41. C. J. Jenvey, J. R. Stabel, Autofluorescence and nonspecific immunofluorescent labeling in frozen bovine intestinal tissue sections: Solutions for multicolor immunofluorescence experiments. *J. Histochem. Cytochem.* **65**, 531–541 (2017).

42. S. Chatterjee, Artefacts in histopathology. *J. Oral. Maxillofac. Pathol.* **18**, S111–S116 (2014).

43. A. Y. Abramov, M. Gegg, A. Grunewald, N. W. Wood, C. Klein, A. H. V. Schapira, Bioenergetic consequences of PINK1 mutations in Parkinson disease. *PLOS ONE* **6**, e25622 (2011).

44. R. Xie, J.-Y. Chung, K. Ylaya, R. L. Williams, N. Guerrero, N. Nakatsuka, C. Badie, S. M. Hewitt, Factors influencing the degradation of archival formalin-fixed paraffin-embedded tissue sections. *J. Histochem. Cytochem.* **59**, 356–365 (2011).

45. J. Hou, H. J. Wright, N. S.-K. Chan, R. D. H. Tran, O. V. Razorenova, E. O. Potma, B. J. Tromberg, Correlating two-photon excited fluorescence imaging of breast cancer cellular redox state with seahorse flux analysis of normalized cellular oxygen consumption. *J. Biomed. Opt.* **21**, 060503 (2016).

46. J. M. Corbin, M. J. Ruiz-Echevarria, One-carbon metabolism in prostate cancer: The role of androgen signaling. *Int. J. Mol. Sci.* **17**, 1208 (2016).

47. L. Zhu, K. Ploessl, R. Zhou, D. Mankoff, H. F. Kung, Metabolic imaging of glutamine in cancer. *J. Nucl. Med.* **58**, 533–537 (2017).

48. J. Jiang, S. Srivastava, J. Zhang, Starve cancer cells of glutamine: Break the spell or make a hungry monster? *Cancers* **11**, 804 (2019).

49. J. Jin, J.-K. Byun, Y.-K. Choi, K.-G. Park, Targeting glutamine metabolism as a therapeutic strategy for cancer. *Exp. Mol. Med.* **55**, 706–715 (2023).

50. A. Varone, J. Xylas, K. P. Quinn, D. Pouli, G. Sridharan, M. E. McLaughlin-Drubin, C. Alonzo, K. Lee, K. Münger, I. Georgakoudi, Endogenous two-photon fluorescence imaging elucidates metabolic changes related to enhanced glycolysis and glutamine consumption in precancerous epithelial tissues. *Cancer Res.* **74**, 3067–3075 (2014).

51. K. M. Holmström, L. Baird, Y. Zhang, I. Hargreaves, A. Chalasani, J. M. Land, L. Stanyer, M. Yamamoto, A. T. Dinkova-Kostova, A. Y. Abramov, Nrf2 impacts cellular bioenergetics by controlling substrate availability for mitochondrial respiration. *Biol. Open* **2**, 761–770 (2013).

52. A. U. Rehman, A. G. Anwer, M. E. Gosnell, S. B. Mahbub, G. Liu, E. M. Goldys, Fluorescence quenching of free and bound NADH in HeLa cells determined by hyperspectral imaging and unmixing of cell autofluorescence. *Biomed. Opt. Express* **8**, 1488–1498 (2017).

53. B. R. Ecclestone, K. Bell, S. Sparkes, D. Dinakaran, J. R. Mackey, P. Haji Reza, Label-free complete absorption microscopy using second generation photoacoustic remote sensing. *Sci. Rep.* **12**, 8464 (2022).

54. J. Jiang, M. Feng, A. Jacob, L. Z. Li, H. N. Xu, *Oxygen Transport to Tissue XLII* (Springer, 2021), pp. 253–258.

55. A. J. Walsh, R. S. Cook, H. C. Manning, D. J. Hicks, A. Lafontant, C. L. Arteaga, M. C. Skala, Optical metabolic imaging identifies glycolytic levels, subtypes, and early-treatment response in breast cancer. *Cancer Res.* **73**, 6164–6174 (2013).

56. A. J. Walsh, J. A. Castellanos, N. S. Nagathihalli, N. B. Merchant, M. C. Skala, Optical imaging of drug-induced metabolism changes in murine and human pancreatic cancer organoids reveals heterogeneous drug response. *Pancreas* **45**, 863 (2016).

57. Z. Liu, D. Pouli, C. A. Alonzo, A. Varone, S. Karaliota, K. P. Quinn, K. Münger, K. P. Karalis, I. Georgakoudi, Mapping metabolic changes by noninvasive, multiparametric, high-resolution imaging using endogenous contrast. *Sci. Adv.* **4**, eaap9302 (2018).

58. A. J. Walsh, R. S. Cook, M. E. Sanders, L. Aurisicchio, G. Ciliberto, C. L. Arteaga, M. C. Skala, Quantitative optical imaging of primary tumor organoid metabolism predicts drug response in breast cancer. *Cancer Res.* **74**, 5184–5194 (2014).

59. A. J. Walsh, K. P. Mueller, K. Tweed, I. Jones, C. M. Walsh, N. J. Piscopo, N. M. Niemi, D. J. Pagliarini, K. Saha, M. C. Skala, Classification of T-cell activation via autofluorescence lifetime imaging. *Nat. Biomed. Eng.* **5**, 77–88 (2021).

60. B. D. Cikaluk, B. S. Restall, N. J. Haven, M. T. Martell, E. A. McAlister, R. J. Zemp, Rapid ultraviolet photoacoustic remote sensing microscopy using voice-coil stage scanning. *Opt. Express* **31**, 10136–10149 (2023).

61. L. Snider, K. Bell, P. Hajireza, R. J. Zemp, Toward wide-field high-speed photoacoustic remote sensing microscopy, in *Photons Plus Ultrasound: Imaging and Sensing 2018* (SPIE, 2018), vol. 10494, pp. 143–150.

62. M. Broekgaarden, A.-L. Bulin, J. Frederick, Z. Mai, T. Hasan, Tracking photodynamic-and chemotherapy-induced redox-state perturbations in 3D culture models of pancreatic cancer: A tool for identifying therapy-induced metabolic changes. *J. Clin. Med.* **8**, 1399 (2019).

63. D. Reichert, L. I. Wadiura, M. T. Erkkilae, J. Gesperger, A. Lang, T. Roetzer-Pejrimovsky, J. Makolli, A. Woehrer, M. Wilzbach, C. Hauger, B. Kiesel, M. Andreana, A. Unterhuber, W. Drexler, G. Widhalm, R. A. Leitgeb, Flavin fluorescence lifetime and autofluorescence optical redox ratio for improved visualization and classification of brain tumors. *Front. Oncol.* **13**, 1105648 (2023).

64. S. You, H. Tu, E. J. Chaney, Y. Sun, Y. Zhao, A. J. Bower, Y.-Z. Liu, M. Marjanovic, S. Sinha, Y. Pu, S. A. Boppart, Intravital imaging by simultaneous label-free autofluorescence-multiharmonic microscopy. *Nat. Commun.* **9**, 2125 (2018).

65. M. A. Ilie, C. Caruntu, M. Lupu, D. Lixandru, M. Tampa, S.-R. Georgescu, A. Bastian, C. Constantin, M. Neagu, S. A. Zurac, D. Boda, Current and future applications of confocal laser scanning microscopy imaging in skin oncology. *Oncol. Lett.* **17**, 4102–4111 (2019).

66. J. M. Campbell, A. Habibalahi, S. Mahbub, M. Gosnell, A. G. Anwer, S. Paton, S. Gronthos, E. Goldys, Non-destructive, label free identification of cell cycle phase in cancer cells by multispectral microscopy of autofluorescence. *BMC Cancer* **19**, 1–11 (2019).

67. M. Wang, H. Z. Kimbrell, A. B. Sholl, D. B. Tulman, K. N. Elfer, T. C. Schlichenmeyer, B. R. Lee, M. Lacey, J. Q. Brown, High-resolution rapid diagnostic imaging of whole prostate biopsies using video-rate fluorescence structured illumination microscopy. *Cancer Res.* **75**, 4032–4041 (2015).

68. J. Mertz, Optical sectioning microscopy with planar or structured illumination. *Nat. Methods* **8**, 811–819 (2011).

69. D. R. Yankelevich, D. Ma, J. Liu, Y. Sun, Y. Sun, J. Bec, D. S. Elson, L. Marcu, Design and evaluation of a device for fast multispectral time-resolved fluorescence spectroscopy and imaging. *Rev. Sci. Instrum.* **85**, 034303 (2014).

70. J. E. Sorrells, R. R. Iyer, L. Yang, E. M. Martin, G. Wang, H. Tu, M. Marjanovic, S. A. Boppart, Computational photon counting using multithreshold peak detection for fast fluorescence lifetime imaging microscopy. *ACS Photonics* **9**, 2748–2755 (2022).

71. L. Shi, C. Zheng, Y. Shen, Z. Chen, E. S. Silveira, L. Zhang, M. Wei, C. Liu, C. de Sena-Tomas, K. Targoff, W. Min, Optical imaging of metabolic dynamics in animals. *Nat. Commun.* **9**, 2995 (2018).

72. Y. Tan, H. Lin, J.-X. Cheng, Profiling single cancer cell metabolism via high-content SRS imaging with chemical sparsity. *Sci. Adv.* **9**, eadg6061 (2023).

73. M. T. Martell, N. J. Haven, B. D. Cikaluk, B. S. Restall, E. A. McAlister, R. Mittal, B. A. Adam, N. Giannakopoulos, L. Peiris, S. Silverman, J. Deschenes, X. Li, R. J. Zemp, Deep learning-enabled realistic virtual histology with ultraviolet photoacoustic remote sensing microscopy. *Nat. Commun.* **14**, 5967 (2022).

74. M. Boktor, B. R. Ecclestone, V. Pekar, D. Dinakaran, J. R. Mackey, P. Fieguth, P. Haji Reza, Virtual histological staining of label-free total absorption photoacoustic remote sensing (TA-PARS). *Sci. Rep.* **12**, 10296 (2022).

75. M. Boktor, J. E. Tweel, B. R. Ecclestone, J. A. Ye, P. Fieguth, P. H. Reza, Multi-channel feature extraction for virtual histological staining of photon absorption remote sensing images. arXiv:2307.01824 [eess.IV] (2023).

76. J. E. Tweel, B. R. Ecclestone, M. Boktor, J. A. T. Simmons, P. Fieguth, P. H. Reza, Virtual histology with photon absorption remote sensing using a cycle-consistent generative adversarial network with weakly registered pairs. arXiv:2306.08583 [physics.med-ph] (2023).

77. F. Fereidouni, Z. T. Harmany, M. Tian, A. Todd, J. A. Kintner, J. D. McPherson, A. D. Borowsky, J. Bishop, M. Lechpammer, S. G. Demos, R. Levenson, Microscopy with ultraviolet surface excitation for rapid slide-free histology. *Nat. Biomed. Eng.* **1**, 957–966 (2017).

78. A. K. Glaser, N. P. Reder, Y. Chen, E. F. McCarty, C. Yin, L. Wei, Y. Wang, L. D. True, J. T. Liu, Light-sheet microscopy for slide-free non-destructive pathology of large clinical specimens. *Nat. Biomed. Eng.* **1**, 1–10 (2017).

79. W. Xie, A. K. Glaser, F. Vakar-Lopez, J. L. Wright, N. P. Reder, J. T. Liu, L. D. True, Diagnosing 12 prostate needle cores within an hour of biopsy via open-top light-sheet microscopy. *J. Biomed. Opt.* **25**, 126502–126502 (2020).

80. Y. K. Tao, D. Shen, Y. Sheikine, O. O. Ahsen, H. H. Wang, D. B. Schmolze, N. B. Johnson, J. S. Brooker, A. E. Cable, J. L. Connolly, J. G. Fijimoto, Assessment of breast pathologies using nonlinear microscopy. *Proc. Natl. Acad. Sci. U.S.A.* **111**, 15304–15309 (2014).

81. L. C. Cahill, Y. Wu, T. Yoshitake, C. Ponchiardi, M. G. Giacomelli, A. A. Wagner, S. Rosen, J. G. Fujimoto, Nonlinear microscopy for detection of prostate cancer: Analysis of sensitivity and specificity in radical prostatectomies. *Mod. Pathol.* **33**, 916–923 (2020).

82. Y. Sun, S. You, X. Du, A. Spaulding, Z. G. Liu, E. J. Chaney, D. R. Spillman Jr., M. Marjanovic, H. Tu, S. A. Boppart, Real-time three-dimensional histology-like imaging by label-free nonlinear optical microscopy. *Quant. Imaging Med. Surg.* **10**, 2177–2190 (2020).

83. D. A. Orringer, B. Pandian, Y. S. Niknafs, T. C. Hollon, J. Boyle, S. Lewis, M. Garrard, S. L. Hervey-Jumper, H. J. Garton, C. O. Maher, J. A. Heth, O. Sagher, D. A. Wilkinson, M. Snuderl, S. Venneti, S. H. Ramkissoon, K. A. Mc Fadden, A. Fisher-Hubbard, A. P. Lieberman, T. D. Johnson, X. S. Xie, J. K. Trautman, C. W. Freudiger, S. Camelo-Piragua, Rapid intraoperative histology of unprocessed surgical specimens via fibre-laser-based stimulated Raman scattering microscopy. *Nat. Biomed. Eng.* **1**, 0027 (2017).

84. T. C. Hollon, B. Pandian, A. R. Adapa, E. Urias, A. V. Save, S. S. S. Khalsa, D. G. Eichberg, R. S. D'Amico, Z. U. Farooq, S. Lewis, P. D. Petridis, T. Marie, A. H. Shah, H. J. L. Garton, C. O. Maher, J. A. Heth, E. L. M. Kean, S. E. Sullivan, S. L. Hervey-Jumper, P. G. Patil, B. G. Thompson, O. Sagher, G. M. Mc Khann II, R. J. Komotar, M. E. Ivan, M. Snuderl, M. L. Otten, T. D. Johnson, M. B. Sisti, J. N. Bruce, K. M. Muraszko, J. Trautman, C. W. Freudiger, P. Canoll, H. Lee, S. Camelo-Piragua, D. A. Orringer, Near real-time intraoperative brain

tumor diagnosis using stimulated Raman histology and deep neural networks. *Nat. Med.* **26**, 52–58 (2020).

85. T. T. Wong, R. Zhang, P. Hai, C. Zhang, M. A. Pleitez, R. L. Aft, D. V. Novack, L. V. Wang, Fast label-free multilayered histology-like imaging of human breast cancer by photoacoustic microscopy. *Sci. Adv.* **3**, e1602168 (2017).

86. T. Imai, J. Shi, T. T. Wong, L. Li, L. Zhu, L. V. Wang, High-throughput ultraviolet photoacoustic microscopy with multifocal excitation. *J. Biomed. Opt.* **23**, 036007 (2018).

87. R. Cao, S. D. Nelson, S. Davis, Y. Liang, Y. Luo, Y. Zhang, B. Crawford, L. V. Wang, Label-free intraoperative histology of bone tissue via deep-learning-assisted ultraviolet photoacoustic microscopy. *Nat. Biomed. Eng.* **7**, 124–134 (2023).

88. L. Kang, X. Li, Y. Zhang, T. T. Wong, Deep learning enables ultraviolet photoacoustic microscopy based histological imaging with near real-time virtual staining. *Photoacoustics* **25**, 100308 (2022).

89. Y. Zhang, L. Kang, I. H. Wong, W. Dai, X. Li, R. C. Chan, M. K. Hsin, T. T. Wong, High-throughput, label-free and slide-free histological imaging by computational microscopy and unsupervised learning. *Adv. Sci.* **9**, 2102358 (2022).

90. S. Soltani, A. Ojaghi, H. Qiao, N. Kaza, X. Li, Q. Dai, A. O. Osunkoya, F. E. Robles, Prostate cancer histopathology using label-free multispectral deep-UV microscopy quantifies phenotypes of tumor aggressiveness and enables multiple diagnostic virtual stains. *Sci. Rep.* **12**, 9329 (2022).

91. M. Ragazzi, S. Piana, C. Longo, F. Castagnetti, M. Foroni, G. Ferrari, G. Gardini, G. Pellacani, Fluorescence confocal microscopy for pathologists. *Mod. Pathol.* **27**, 460–471 (2014).

92. J. Pérez-Anker, S. Ribero, O. Yélamos, A. Garcia-Herrera, L. Alos, B. Alejo, M. Combalia, D. Moreno-Ramirez, J. Malvehy, S. Puig, Basal cell carcinoma characterization using fusion

ex vivo confocal microscopy: A promising change in conventional skin histopathology. *Br. J. Dermatol.* **182**, 468–476 (2020).

93. J. Li, J. Garfinkel, X. Zhang, D. Wu, Y. Zhang, K. De Haan, H. Wang, T. Liu, B. Bai, Y. Rivenson, G. Rubinstein, P. O. Scumpia, A. Ozcan, Biopsy-free in vivo virtual histology of skin using deep learning. *Light Sci. Appl.* **10**, 233 (2021).

94. B. E. Bejnordi, N. Timofeeva, I. Otte-Höller, N. Karssemeijer, J. A. van der Laak, Quantitative analysis of stain variability in histology slides and an algorithm for standardization, in *Medical Imaging 2014: Digital Pathology* (International Society for Optics and Photonics, 2014), vol. 9041, pp. 904108.

95. K. B. Patel, W. Liang, M. J. Casper, V. Voleti, W. Li, A. J. Yagielski, H. T. Zhao, C. Perez Campos, G. S. Lee, J. M. Liu, E. Philipone, A. J. Yoon, K. P. Olive, S. M. Coley, E. M. C. Hillman, High-speed light-sheet microscopy for the in-situ acquisition of volumetric histological images of living tissue. *Nat. Biomed. Eng.* **6**, 569–583 (2022).

96. T. Yoshitake, M. G. Giacomelli, L. M. Quintana, H. Vardeh, L. C. Cahill, B. E. Faulkner-Jones, J. L. Connolly, D. Do, J. G. Fujimoto, Rapid histopathological imaging of skin and breast cancer surgical specimens using immersion microscopy with ultraviolet surface excitation. *Sci. Rep.* **8**, 1–12 (2018).

97. W. Xie, Y. Chen, Y. Wang, L. Wei, C. Yin, A. K. Glaser, M. E. Fauver, E. J. Seibel, S. M. Dintzis, J. C. Vaughan, N. P. Reder, J. T. C. Liu, Microscopy with ultraviolet surface excitation for wide-area pathology of breast surgical margins. *J. Biomed. Opt.* **24**, 026501 (2019).

98. E. Olson, M. J. Levene, R. Torres, Multiphoton microscopy with clearing for three dimensional histology of kidney biopsies. *Biomed. Opt. Express* **7**, 3089–3096 (2016).

99. Y. Rivenson, H. Wang, Z. Wei, K. de Haan, Y. Zhang, Y. Wu, H. Günaydın, J. E. Zuckerman, T. Chong, A. E. Sisk, L. M. Westbrook, W. D. Wallace, A. Ozcan, Virtual histological staining of unlabelled tissue-autofluorescence images via deep learning. *Nat. Biomed. Eng.* **3**, 466–477 (2019).

100. Y. Winetraub, E. Yuan, I. Terem, C. Yu, W. Chan, H. Do, S. Shevidi, M. Mao, J. Yu, M. Hong, E. Blankenberg, K. E. Rieger, S. Chu, S. Aasi, K. Y. Sarin, A. de la Zerda, OCT2Hist: Non-invasive virtual biopsy using optical coherence tomography. medRxiv 2021.03.31.21254733 [Preprint] (2021). https://doi.org/10.1101/2021.03.31.21254733.

101. D.-K. Yao, K. Maslov, K. K. Shung, Q. Zhou, L. V. Wang, In vivo label-free photoacoustic microscopy of cell nuclei by excitation of DNA and RNA. *Opt. Lett.* **35**, 4139–4141 (2010).

102. T. T. Wong, R. Zhang, C. Zhang, H.-C. Hsu, K. I. Maslov, L. Wang, J. Shi, R. Chen, K. K. Shung, Q. Zhou, L. V. Wang, Label-free automated three-dimensional imaging of whole organs by microtomy-assisted photoacoustic microscopy. *Nat. Commun.* **8**, 1386 (2017).

103. H. Kim, J. W. Baik, S. Jeon, J. Y. Kim, C. Kim, PAExM: Label-free hyper-resolution photoacoustic expansion microscopy. *Opt. Lett.* **45**, 6755–6758 (2020).

104. X. Li, L. Kang, Y. Zhang, T. T. W. Wong, High-speed label-free ultraviolet photoacoustic microscopy for histology-like imaging of unprocessed biological tissues. *Opt. Lett.* **45**, 5401–5404 (2020).

105. C. Zhang, Y. S. Zhang, D.-K. Yao, Y. Xia, L. V. Wang, Label-free photoacoustic microscopy of cytochromes. *J. Biomed. Opt.* **18**, 020504 (2013).

106. V. Gorti, F. E. Robles, Characterizing UV-induced photo-damage to improve long-term, dynamic live cell imaging with UV microscopy, in *Label-free Biomedical Imaging and Sensing (LBIS) 2024* (SPIE, 2024), vol. 12854, pp. 43–46.

107. L. Shi, L. Lu, G. Harvey, T. Harvey, A. Rodriguez-Contreras, R. R. Alfano, Label-free fluorescence spectroscopy for detecting key biomolecules in brain tissue from a mouse model of Alzheimer's disease. *Sci. Rep.* **7**, 2599 (2017).
